# GFAP and UCH-L1 for Ruling out Intracranial Lesions After Mild Traumatic Brain Injury: A Systematic Review and Meta-Analysis

**DOI:** 10.3390/jcm15134858

**Published:** 2026-06-23

**Authors:** Lorena San Miguel, Vicky Jespers, Dominique Roberfroid

**Affiliations:** Belgian Health Care Knowledge Centre (KCE), Doorbuilding, Boulevard du Jardin Botanique 55, 1000 Bruxelles, Belgium

**Keywords:** biomarkers, diagnostic accuracy, traumatic brain injuries, intracranial injuries, CT scan

## Abstract

**Background**: Patients with mild traumatic brain injury (mTBI) have a small but clinically relevant risk of intracranial injury (ICI), requiring timely detection. Computed tomography (CT) remains the diagnostic gold standard but is costly and exposes patients to ionising radiation. Combining blood-based biomarkers, glial fibrillary acidic protein (GFAP) and ubiquitin carboxy-terminal hydrolase L1 (UCH-L1), with clinical decision rules may allow safe exclusion of ICI without CT, reducing unnecessary imaging, radiation exposure, and resource use. **Methods**: A systematic review of clinical and economic studies in patients with mTBI was registered in PROSPERO (CRD420251051158). Searches were conducted in January 2025 and updated in May 2025 in MEDLINE, Embase, and the Cochrane Library. The aim was to assess the diagnostic accuracy and economic value of the combination of GFAP and UCH-L1 compared with CT scanning to rule out ICI in both adults and children with mTBI. Where available, studies directly comparing GFAP and UCH-L1 with S100β were also analysed descriptively. The quality of the clinical evidence was assessed with QUADAS-2 and GRADE. Meta-analyses used a bivariate random-effects model, with heterogeneity and sensitivity analyses explored. **Results**: Overall, 21 studies were considered in our review. Moderate- to high-quality evidence indicates that GFAP and UCH-L1, when used together with clinical assessment, have very high sensitivity and can reliably rule out ICI in adults with mTBI presenting within 12 h to the emergency department. Evidence for paediatric populations shows promise but remains very limited. Specificity is low, particularly in older adults, which limits the ability to reduce CT use in this high-risk group. Research on age-adjusted cut-offs is ongoing and may help to reduce the proportion of false positive tests without compromising sensitivity. Few studies directly compared GFAP and UCH-L1 with S100β, with slightly higher to equivalent sensitivity for GFAP and UCH-L1. Economic evaluations suggest possible cost savings and reduced CT utilisation, but these analyses rely on assumptions unsupported by robust data and are highly context-dependent. There is a lack of clarity in the included studies regarding whether existing clinical head rules were used to define the study populations (i.e., to determine which patients would be recommended for CT scanning) and, if so, which specific rules were applied. **Conclusions**: Evidence shows that GFAP and UCH-L1 can safely exclude ICI in adults with mTBI in whom a CT scan would otherwise be considered based on clinical assessment or decision rules. Nevertheless, real-world evidence and cost-effectiveness data are scarce. Further prospective studies, including paediatric and elderly populations, and integration with clinical decision rules will be informative to ensure optimal use in clinical practice.

## 1. Introduction

Traumatic brain injuries (TBI) are non-degenerative, non-congenital insults to the brain from an external mechanical force, which may lead to a temporary or permanent impairment of cognitive, physical, and psychosocial functions. Falls are the leading cause of TBI in high-income countries, particularly among older people (≥65 years), followed by motor vehicle accidents, and blows to the head [1].

TBI represents a substantial public health problem around the world [2], with a global estimated age-standardised incidence rate of TBI in 2019 of 369 per 100,000 population (95%CI: 331–412) [3].

TBI severity is commonly classified as mild, moderate, or severe based on the Glasgow Coma Scale (GCS), which ranges from 3 to 15 and assesses the level of consciousness and neurological function after head injury.

Mild TBI, typically defined as GCS 13–15 [2], accounts for approximately 90% of all TBI cases [1,2,4].

Although the prognosis for mTBI is generally favourable, patients remain at risk of clinically important complications: ICIs occur in around 7% of cases, while lesions leading to death or requiring neurosurgical intervention occur in about 0.9% (95% CI, 0.78–1.0%) [5].

CT scanning is the gold standard for detecting ICI due to its high sensitivity and availability. However, it is costly and exposes patients to radiation, which is of particular concern in children and young adults [6,7,8].

Clinical decision rules, such as the Canadian CT Head (CCTH) rule or the New Orleans Criteria for adults or PECARN for children, aim to limit unnecessary imaging in mTBI. Despite a general high sensitivity and negative predictive value, enabling a reduction in CT use of around 30–40% [5,9,10,11,12,13], many patients without ICI still undergo scanning [5,10,13]. Emerging biomarkers may help further reduce avoidable CT imaging.

Several blood biomarkers have been investigated in the management of mTBI in emergency departments, particularly to rule out ICIs. A CT scan is no longer indicated if the biomarker test result is negative, and the patient can be discharged with instructions on what to do if symptoms occur. If the biomarker tested is positive, CT imaging is performed.

Three biomarkers have received the most attention: S100β, which has been the most extensively studied [14,15,16], while research on glial fibrillary acidic protein (GFAP) and ubiquitin C-terminal hydrolase-L1 (UCH-L1) is more recent. This article evaluates the performance of a combination of two such biomarkers that have gained increasing attention in recent years: glial fibrillary acidic protein (GFAP) and ubiquitin C-terminal hydrolase-L1 (UCH-L1).

GFAP, an intermediate filament protein mainly produced by astrocytes, is released into cerebrospinal fluid after astrocytic injury and subsequently reaches the bloodstream. It has a 24–48 h half-life, rises within 1 h, peaks at approximately 20 h, and declines over 1–2 days.

UCH-L1, a neuronal enzyme involved in proteasomal degradation, also appears in the blood after brain injury, although the exact pathways remain uncertain. Its half-life is much shorter (6–7 h), becoming detectable within 30 min, peaking at approximately 8 h, and rapidly decreasing thereafter.

Interest in combining these two proteins has increased in recent years due to their complementary temporal profiles. UCH-L1 reaches peak concentrations soon after mTBI, allowing early ICI detection, whereas GFAP peaks later, enabling identification of patients with ICIs several hours after trauma. Together, these biomarkers extend the time-window for use to 12 h post-injury.

Combined measurement of GFAP and UCH-L1 is currently available on three commercial FDA-approved clinical platforms:

- i-STAT Alinity point-of-care handheld analyser (electrochemiluminescence; Abbott; Chicago, IL, USA),
- Alinity i TBI core laboratory analyser (chemiluminescent microparticle immunoassay, CMIA; Abbott; Chicago, IL, USA), and
- VIDAS TBI platform (enzyme-linked fluorescent assay, bioMérieux; Lyon, France).

The test result is considered negative if both GFAP and UCH-L1 concentrations are below the predefined cut-offs, whereas a positive result is defined by at least one biomarker concentration exceeding its predefined cut-off.

This review evaluates the diagnostic accuracy and economic impact of combined GFAP and UCH-L1 testing to rule out ICIs after mTBI and to reduce unnecessary CT imaging among low-risk patients. In addition, available direct comparisons between GFAP and UCH-L1 and S100β are reported for a more complete view.

## 2. Materials and Methods

### 2.1. Clinical Review

We conducted a PROSPERO-registered systematic review (CRD420251051158) following PRISMA-DTA to assess the diagnostic accuracy of GFAP and UCH-L1 for ruling out ICI in mTBI using CT/MRI as reference standards. Systematic searches in MEDLINE, Embase, and the Cochrane Library, as well as consultation of HTA agency websites, were performed in January 2025 and updated in May 2025 to identify relevant diagnostic accuracy studies. Data were extracted on sensitivity, specificity and positive (LR+) and negative (LR-) likelihood ratios, and their quality was assessed with QUADAS-2 (study-level) [17] and GRADE (outcome-level) [18,19]. When reported, data on S100β measured within the same studies were also extracted to allow direct comparison with GFAP and UCH-L1.

Meta-analyses using a bivariate random-effects model estimated pooled accuracy, explored heterogeneity and generated summary ROC curves.

Sensitivity analyses examined biomarker performance by analyser type, age, cut-off thresholds, timing of sampling, and study quality. All analyses were performed using Stata 15.0.

### 2.2. Economic Review

A specific systematic search on costing studies was conducted on 14 July 2025, across MEDLINE, Embase, EconLit, and the Cochrane Library to identify primary full economic evaluations (comparing costs and outcomes) and secondary research (systematic reviews of economic evaluations). Additionally, we screened Health Technology Assessment agency websites listed on the International Network of Agencies for Health Technology Assessment (INAHTA) to capture relevant reports. No language restrictions were applied. Study selection and data extraction were performed individually but any doubts were discussed with a second reviewer. Studies finally included in our review were critically appraised using an in-house structured data extraction sheet based on the CHEERS 2022 checklist (https://www.equator-network.org/wp-content/uploads/2013/04/CHEERS-2022-checklist-1.pdf, accessed on 1 September 2025).

The complete search strategies and additional details on methods used are provided in the Supplementary Materials.

## 3. Results Clinical Review

After removal of duplicates, a total of 1787 records were screened, and 18 primary studies were included after title/abstract and full-text assessment. Reference lists of included studies and five relevant systematic reviews (SRs) identified during the search were also screened to ensure complete study capture.

**Figure 1:**
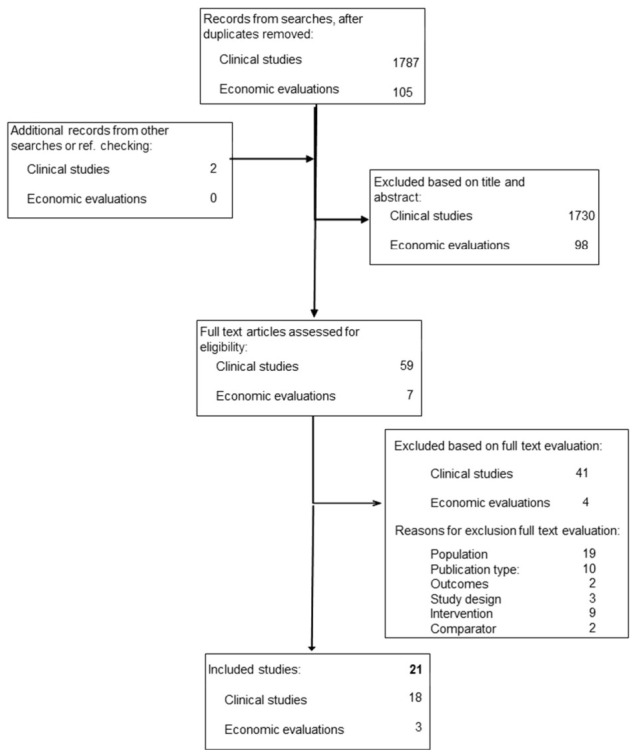
PRISMA flow chart for clinical and economic study selection.

Our reasons for full-text exclusion are provided in the Supplementary Materials (see Table S2.3), and the study selection process is summarised in a PRISMA flow diagram (see Figure 1).

Of the included studies, 17 focused on adults, and only one specifically examined a paediatric population [20]. One study was limited to older adults [21], and three others provided subgroup data for individuals aged ≥65 years. In Ladang 2025, the percentage of adults <65 years and ≥65 years with a positive CT scan was provided by the authors at our request, which allowed the inclusion of their results in the meta-analysis stratified by age group [22]. Additional study information and results, including mTBI definition, assay type, blood matrix (serum and/or plasma), biomarker cut-offs, timing of sampling, incidence of CT-confirmed ICIs, and key diagnostic accuracy metrics are presented in Table 1. Only results pertaining to mTBI were extracted from studies with mixed TBI severity populations.

Of the 18 studies retrieved, three were excluded from meta-analysis. Trivedi 2024 lacked defined biomarker cut-off values [36]. Harris 2025 did not report the proportion of GCS 13–15 patients with positive CT findings [27]. Puravet 2025 included children only (<16 years), and applied age-specific GFAP and UCH-L1 cut-offs, whereas all other studies focused on adults [20]. A narrative description of the results of Puravet 2025 is provided later on in this article under the section on results by age [20].

Thus, fifteen adult studies were finally retained for the meta-analysis.

### 3.1. General Performance of GFAP and UCH-L1 in Adults

A table describing the general characteristics of these studies is provided as a supplement (see Table S2.4). The use of a specific validated head rule to select patients eligible for a CT scan was reported in only five studies [21,25,29,33,34]. This does not necessarily imply that the other studies did not use a clinical algorithm or structured assessment; rather, they did not specify how patients with mTBI requiring a CT scan were selected.

The cut-offs defining a positive test were predefined in studies using the i-STAT Alinity (Abbott), the Alinity i platform (Abbott), or the VIDAS (bioMérieux) (see Table 1). In other studies, the cut-offs were either close to predefined manufacturer cut-offs or substantially different. In case more than one set of cut-offs was tested, we included in the primary meta-analysis the set yielding the lowest LR-, and tested the alternative set in a sensitivity analysis.

**Figure 2:**
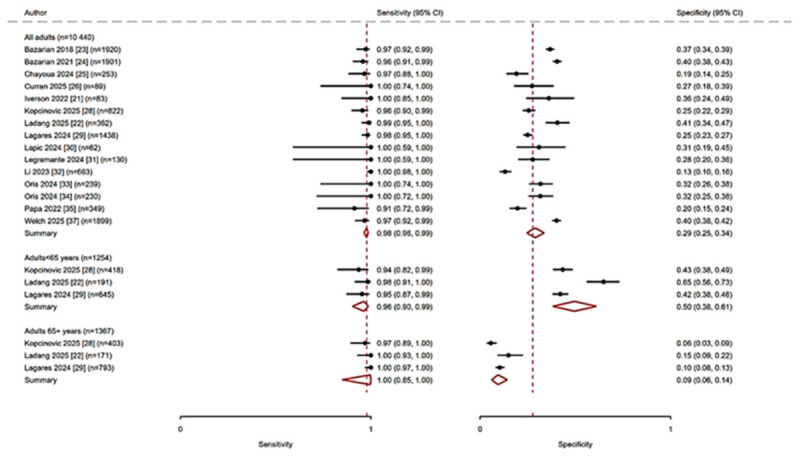
Forest plot of all studies and per age group.

The pooled sensitivity and specificity over the 15 studies (n = 10,440 adults) were 98% (95%CI: 96–99; I^2^ = 4.3%) and 29% (95%CI: 25–34; I^2^ = 85.6%), respectively. The LR- was 0.08 (95%CI: 0.05–0.12), i.e., the power of a negative result to rule out a brain lesion was very high. However, the reduction in CT scan was highly dependent on the specificity, which varied considerably across studies (I^2^ = 85.6%) as shown in the forest plot (Figure 2).

Ladang 2025 [22], provided the percentage of patients with a CT scan positive in adults <65 years and adults ≥65 years at our request, which allowed the inclusion of their results in the meta-analysis stratified by age group. For Li 2023 [32], results for the ELISA assays on plasma or serum were merged, as results were very similar.

The marked heterogeneity in specificity across studies could partly be explained by the age of participant populations, as shown in the subgroup analysis. For some studies, the confidence intervals around the sensitivity were wide, e.g., the lower bound was 74% in Curran 2025 [26] and 59% in Lapić 2024 [30]. This imprecision was primarily due to small sample sizes (e.g., 89 in Curran 2025 [26], 62 in Lapić 2024 [30]), and therefore, their impact on the pooled estimate and I^2^ was limited.

It is important to highlight that in the few studies with false negative cases, none of these was linked to lesions requiring immediate neurosurgery, i.e., the consequence of a false negative test did not jeopardise the survival of the patients. However, the diagnoses of false negative tests were not reported in three studies [28,35,37]. More details are provided in Table 2.

### 3.2. Results by Analyser

Various types of analysers were used: the handheld i-STAT Alinity (Abbott) in three studies [24,25,34], the Alinity i platform (Abbott) in seven studies [22,26,28,30,31,33,37], and VIDAS (bioMérieux) in only one study [29]. The remaining four studies used various ELISA assays or digital immunoassays. We assessed if the performance of GFAP and UCHL-1 varied by device. As can be seen in the stratified forest plot, no major differences in pooled sensitivity were apparent, with very similar point estimates and overlapping confidence intervals (see Figure S2.5 in the Supplementary Materials). In contrast, the specificity of ELISA assays and VIDAS appeared to be lower, by about 5 to 8 percentage points, in comparison with i-STAT Alinity and Alinity I, but confidence intervals were also overlapping.

### 3.3. Results by Age

Results stratified by age were provided in three studies [22,28,29]. The forest plot (see Figure 2) shows that sensitivity was barely affected by age. In contrast, the specificity was much lower in adults aged ≥ 65 years (9%; 95%CI: 6–14) than in adults aged < 65 years (50%; 95%CI: 38–61). A fourth study [21] was not meta-analysed because it included only adults aged ≥ 60 years. Although sensitivity was also high (100%), specificity was greater than in the three studies with adults aged ≥ 65 years (36%; 95%CI: 24.2–49.4). The reason for this higher specificity is unclear, but could be linked to the use of a different assay (Simoa digital immunoassay) and different cut-offs (323 ng/L GFAP, 42 ng/L UCH-L1) than the ones recommended by the manufacturer for the Alinity i platform, which were used in the other three studies.

The only study conducted on children (<16 years) was published by Puravet et al. in 2025 [20]. The authors applied age-specific GFAP and UCH L1 cut-offs (Table 1). They included 531 children with a GCS score of 15 who required hospitalisation or head CT according to French Paediatric Society guidelines (i.e., the intermediate risk group of the PECARN algorithm). Of these, 68 underwent CT imaging.

Diagnostic performance was first assessed by defining the test as positive when both GFAP and UCH L1 exceeded their respective thresholds. Under this definition, sensitivity was 100% (95% CI: 75–100) and specificity was 42% (95% CI: 29–56). A further analysis was performed using an alternative definition, in which the test was considered positive if either biomarker exceeded its threshold. Sensitivity remained 100%, but specificity decreased substantially to 13% (95% CI: 5–25).

### 3.4. Results by Cut-Off

Some studies investigated how varying the cut-offs could improve the performance of the test. Ladang et al. 2025 [22] compared the cut-offs recommended by the manufacturer for the Alinity i platform (35 ng/L for GFAP, 400 ng/L for UCH-L1) to age-dependent cut-offs, i.e., in adults ≥ 65 years, the new cut-offs were 115 ng/L for GFAP and 335 ng/L for UCH-L1, whereas the usual cut-offs were maintained in adults < 65 years. In adults ≥ 65 years, the specificity increased from an initial 15% to 31%, and in the whole group (all adults), specificity improved from 41% to 48%, without decreasing sensitivity. The number of CT scans avoided was thus doubled in adults ≥ 65 years. In Papa 2022 [35], cut-offs of 30 ng/L for GFAP and 327 ng/L for UCH-L1 were used for their ELISA assay, i.e., close to the cut-offs recommended for the i-STAT Alinity (Abbott). They yielded a sensitivity of 91% (95%CI: 72–99) and a specificity of 20% (95%CI: 15–24). When the cut-offs of 67 ng/L GFAP and 189 ng/L UCH-L1 were used, the sensitivity increased to 100% (95%CI: 82–100) and specificity went up to 25% (95%CI: 20–30) [35]. These two examples show that the specificity of the test can be improved by increasing the cut-off for GFAP while preserving sensitivity through a reduction in the UCH-L1 cut-off.

### 3.5. Results by Time from Trauma to Sampling

Regarding the time elapsed between trauma and blood sampling, the vast majority of the studies aimed at a maximum time of 12 h, and when the actual median time was reported, it was less than 6 h in most studies (see Table 1). Because of this relative homogeneity across studies, assessing the effect of this parameter on the performance of the test was not possible. In Oris 2024 [33], the sensitivity (around 100%) and the specificity (around 30%) remained very similar when the blood samples were collected within either 3 or 12 h after the trauma, which was not unexpected, given that 80% of samples obtained within 12 h were collected within the first 3 h.

### 3.6. Comparison with S100β

A comparison of the combination of GFAP and UCH-L1 with the protein S100β was provided in five of the studies identified via our review (a summary table is presented in Table S2.8 in the Supplementary Materials) [20,29,32,33,36]. In Puravet 2025, this was carried out in children [20] and Trivedi 2024 [36] determined the cut-offs to maximise the Youden’s index. In two of the three remaining studies (i.e., Lagares 2024 [29] and Li 2023 [32]), the protein S100β (measured within 6 h) had a lower sensitivity than GFAP and UCH-L1. The sensitivity of protein S100β (measured within 3 h) and that of GFAP and UCH-L1 were similar in the last study by Oris 2024 [33], but the specificity was lower for the protein S100β (26% vs. 32%), although this difference was not statistically significant.

### 3.7. Quality of the Studies

**Figure 3:**
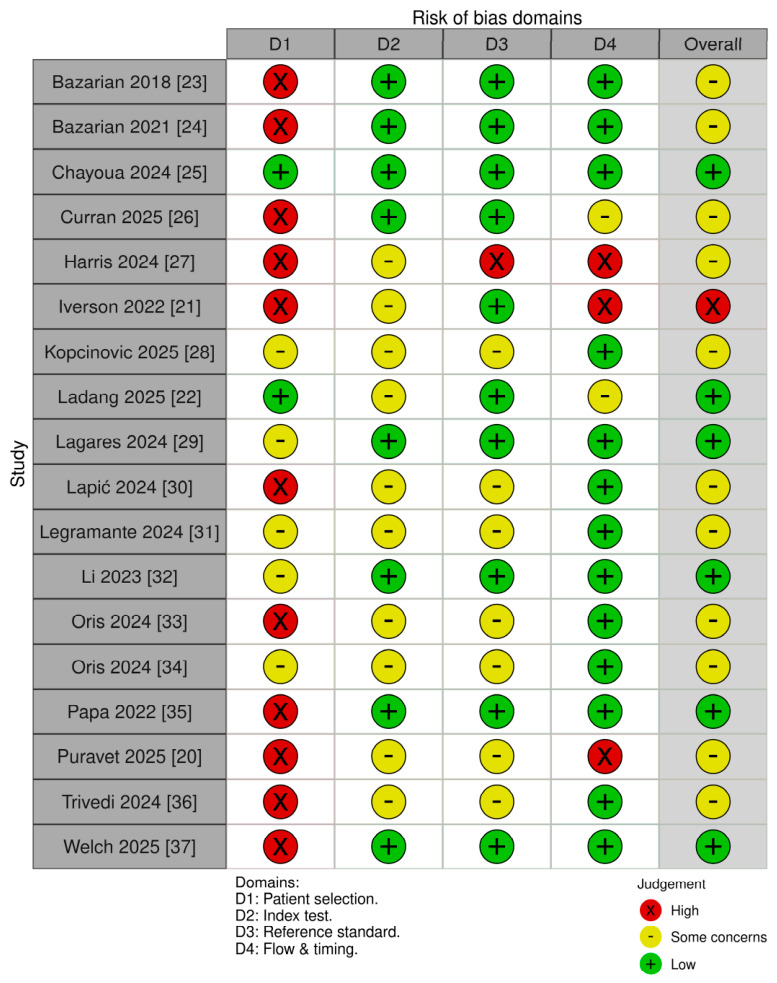
Risk of bias for included studies.

**Figure 4:**
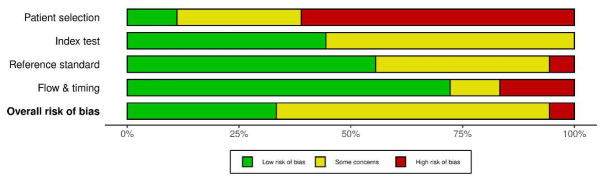
Summary plot QUADAS—2.

The quality of the studies was also relatively homogeneous, and stratified analyses could therefore not be performed. Over the 15 studies included, six studies were considered at low risk of bias, eight studies presented some concerns, and only one was considered to be at high risk of bias. The results for each included study are summarised in Figure 3 and an overview is presented in Figure 4.

No publication bias was apparent in the Deeks’ funnel plot (*p* = 0.16), as shown in Figure S2.9 in the Supplementary Materials.

Following the GRADE framework, a one-level reduction in the quality of evidence could be considered for indirectness, as the biomarkers were not tested in real-world practice studies. Furthermore, the quality of evidence could be lowered by an additional level for specificity due to the significant heterogeneity observed between studies. The level of evidence would therefore be moderate to high for sensitivity, and low to moderate for specificity.

## 4. Results Review of the Costing Studies

Our search of costing studies identified three model-based economic evaluations assessing the use of GFAP and UCH-L1 biomarkers to rule out ICI in patients with mTBI [38,39,40]. Figure 1 shows the flow chart for study selection. Two studies were cost-utility analyses (CUAs) conducted in the USA and France [38,39], and one was a budget impact analysis (BIA) from Spain [40]. All relied on the ALERT-TBI study for biomarker accuracy (sensitivity ~97.3%, specificity ~36.7%, LR- ~0.07) and focused on adult populations presenting to the emergency department with suspected mTBI (GCS 14–15) [23]. Across evaluations, biomarker-guided strategies consistently reduced CT scan utilisation compared to standard care. The Spanish BIA reported the largest impact, with a 35% reduction in overall CT scans and estimated savings of €199 per patient (€899 for standard care vs. €700 for biomarker strategy), alongside shorter patient management times [40]. The French CUA found a 30% reduction in CT scans but only modest savings (€4.15 per patient) [38], while the US study suggested that a biomarker-first strategy could be cost-effective at a willingness-to-pay threshold of US$50,000/QALY if the cost of the test does not exceed US$308 [39]. Gains in quality-adjusted life years (QALYs) were negligible across all diagnostic options, reflecting the primary role of biomarkers in reducing imaging utilisation rather than improving health outcomes. One economic evaluation compared GFAP and UCH-L1 with S100β and found similar health outcomes (QALYs), with a modest additional reduction in CT scans and lower costs for the GFAP and UCH-L1 strategy [38]. Results are presented in Table 3.

Important limitations remain. All studies were based on strong assumptions about clinical practice patterns and relied on a single source for biomarker accuracy. Only one model clearly incorporated clinical decision rules, even though biomarkers are intended as adjuncts rather than replacements for these tools. Real-world implementation challenges, such as workflow integration, turnaround time, and test availability, were not addressed. Furthermore, differences in healthcare tariffs and follow-up imaging practices contributed to variability in estimated savings, limiting transferability to other settings such as Belgium.

## 5. Discussion

The primary objective of the biomarker-based test is to rule out cerebral lesions. To achieve this aim, the highest sensitivity and the lowest LR- are required. Available studies reported a good performance of GFAP and UCH-L1, without major variations across subgroup analyses. The near-100% sensitivity is due to the different and complementary half-lives of the two proteins. It should be noted that most of the studies were carried out on adults, and more research is needed in the paediatric population, in whom avoiding unnecessary CT scanning is particularly important. Nevertheless, the only study found on children shows promising results and new studies on this population are ongoing (Table S2.6 in the Supplementary Materials).

In contrast to sensitivity, the specificity of the test was overall quite low (29%; 95%CI: 25–34), which results in a substantial proportion of tests being false positives, particularly if the pre-test probability of a cerebral lesion is low. The specificity is even lower in adults aged over 65, likely reflecting higher baseline biomarker levels associated with neurodegeneration and ageing. Age-stratified reference ranges may be necessary for accurate interpretation, as demonstrated in the study by Ladang et al., where the number of CT scans avoided in adults ≥ 65 years doubled when the cut-offs for that sub-population were modified [22,41]. However, further validation is necessary before such age-specific thresholds can be established. To date, the extent to which CT use can be safely reduced in older populations remains uncertain. New research should focus on identifying methods to improve the specificity of the GFAP and UCH-L1 test without sacrificing its sensitivity. Direct comparisons between the combination of GFAP+UCH-L1 and the protein S100β remain limited in number and heterogeneous in design. Therefore, the incremental value of one over the other cannot be definitively established within short time windows after trauma. However, when the time window exceeds the 3–6 h from trauma to sampling, S100β is no longer recommended.

In addition, the available economic evaluations rely on several assumptions mainly regarding clinical management pathways (e.g., patient disposition following negative biomarker or CT results), which vary across settings and introduce uncertainty in the estimated cost savings.

It is important to highlight that biomarkers are not intended as standalone tools but as a complement to clinical assessment or decision rules, particularly in patients with mTBI for whom CT would otherwise be considered, because ICI cannot be safely excluded. They are not applicable to patients with clear indications for CT, where results would not alter management.

It should also be noted that research so far has focused on the ability of biomarkers to rule out brain injury that would be detectable by a CT scan. In that context, elevated biomarker concentrations with a negative CT scan are considered a failure of the test (false positive). It has been suggested that blood biomarkers are more objective and sensitive than head CT and they might be able to detect subtler ICIs not visible with standard neuroimaging techniques, but detectable using MRI. A positive test may reflect microstructural lesions which are not detectable at a macrostructural level with CT scanning. Detecting such microstructural lesions could serve as a guide to more structured follow-up, aiming to reduce long-term (persisting) symptoms [4,23,25]. The inadequacy of head CT scan as a reference method to detect microstructural lesions may partly explain the low specificity values of biomarkers [42]. Therefore, more research is also needed on the possible association between elevated biomarkers and longer-term health outcomes. For example, one study on moderate-to-severe TBI showed that early blood-based biomarkers such as GFAP and UCH-L1 can improve prediction of ICI and clinical outcomes compared with clinical variables alone, supporting the potential added value of objective biological markers across the TBI spectrum [43].

## 6. Conclusions

Current (moderate to high-quality) evidence shows that combined GFAP and UCH-L1 testing can reliably exclude ICI in adults with mTBI presenting within 12 h. However, specificity is reduced in older adults, limiting potential reductions in CT use in this specific population. Age-adjusted thresholds may address this but require validation to ensure improved specificity without loss of sensitivity. Evidence in paediatric populations remains insufficient, with only one study identified, precluding clinical implementation at present. Limited comparative evidence suggests that this combination performs at least as well as S100β in terms of sensitivity, within the short time frames from trauma to sampling recommended for the protein S100β.

Real-world implementation data remain scarce and of low quality. While preliminary findings indicate a potential 30% reduction in unnecessary CT scans and modest cost savings, these estimates rely on limited and context-dependent evidence. Robust studies using real-world data and integrating clinical decision rules are needed to confirm clinical utility and cost-effectiveness across diverse healthcare settings.

To our knowledge, this study represents the largest meta-analysis on this topic to date and is the first to jointly synthesise available evidence on diagnostic accuracy and economic implications.

## Figures and Tables

**Table 1:** Performance of GFAP and UCH-L1 to rule out cerebral injuries.

Study	Population	Cut-Offs GFAP and UCH-L1 (pg/mL)	Median Time Measured from Injury in Hours	Measured Within ‘Hours’	Analyser (Provider)	N	CT+ %	Sensitivity % (95% CI)	Specificity % (95% CI)	LR-
Bazarian 2018 [23]	Adults. Mean age 48.8 GCS: 14–15	22 and 327	3.2	12	Chemiluminescent sandwich ELISA in serum (Banyan)	1920	5.9	97.3 (92.4; 99.4)	36.7 (34.5; 39.0)	0.07 (0.00; 0.16)
Bazarian 2021 [24]	Adults mean age 49.1. GCS: 13–15	30 and 360	3.1	12	i-STAT Alinity in plasma (Banyan)	1901	6.3	95.8 (90.6; 98.2)	40.4 (38.2; 42.7)	0.10 (0.04; 0.23)
Chayoua 2024 [25]	Adults. Median age: 48. GCS: 13–15	30 and 360	Most within 3 h	24	i-STAT Alinity in plasma (Abbott)	253	23.3	97 (89; 99)	19 (14; 25)	0.18 (0.04; 0.72)
Curran 2025 [26]	Adults. Mean age: 63. GCS: 13–15	35 and 400	-	12	Alinity i platform in plasma/serum (Abbott)	89	13.5	100 (76; 100)	27 (19; 38)	0.0
Harris 2025 [27]	Patients aged ≥17 GCS: 13–15	30 and 360	-	24	i-STAT Alinity in plasma (Abbott) ARCHITECT platform (Abbott) (I-STAT Alinity and ARCHITECT are two highly correlated assays, and ARCHITECT values were converted to i-STAT equivalents using previously derived equations.)	599	NA	97 (94; 99)	24 (20; 29)	0.12 (0.05; 0.27)
Iverson 2022 [21]	Aged ≥60. Mean age: 79 GCS: 13–15.	323 and 42 AUC	3.1	12	Simoa digital immunoassay in serum (Quanterix)	83	26.5	100 (85.0; 100)	36.1 (24.2; 49.4)	0
Kopcinovic 2025 overall [28]	Adults. Median age: 64 GCS: 13–15	35 and 400	-	12	Alinity i platform in plasma/serum (Abbott)	822	13.6	95.5 (89.9; 98.5)	25.5 (22.3; 28.9)	0.18 (0.07; 0.42)
Kopcinovic 2025 <65 years [28]	Adults aged <65 GCS: 13–15	35 and 400	-	12	Alinity i platform in plasma/serum (Abbott)	418	11.2	93.6 (82.5; 98.7)	43.4 (38.3; 48.6)	0.15 (0.05; 0.44)
Kopcinovic 2025 ≥65 years [28]	Adults aged ≥65 GCS: 13–15	35 and 400	-	12	Alinity i platform in plasma/serum (Abbott)	403	16.1	96.2 (89.3; 99.6)	5.6 (3.4; 8.6)	0.06 (0.01; 0.22)
Ladang 2025 overall [22]	Adults. Median age: 64 GCS: 13–15	35 and 400	-	12	Alinity i platform in plasma (Abbott)	362	31.2	99.1 (95.2–99.98)	40.6 (34.4; 46.9)	0.02 (0.00; 0.15)
Ladang 2025 <65 years [22]	Adults aged <65 GCS: 13–15	35 and 400	-	12	Alinity i platform in plasma (Abbott)	191	33.0	98 (91–100)	65 (56–73)	0.02 (0.00; 0.17)
Ladang 2025 ≥65 years [22]	Adults aged <65. GCS: 13–15	35 and 400	-	12	Alinity i platform in plasma (Abbott)	171	29.2	100 (93–100)	15 (9–22)	0
Ladang 2025 overall, modified cut-offs in ≥65 years [22]	Adults. Median age: 64. GCS: 13–15	Cut-offs in 65+: 115 and 335	-	12	Alinity i platform in plasma (Abbott)	362	31.2	99.1	48.2	0.02 (0.00; 0.13)
Lagares 2024 overall [29]	Adults ≥18 y in FR and ≥15 y in Sp. Median age 69. GCS: 13–15	22 and 327	4.5	12	VIDAS in serum (bioMérieux)	1438	12.2	98.3 (95; 99.7)	24.9 (22.6; 27.4)	0.07 (0.02; 0.21)
Lagares 2024 <65 years [29]	Adults <65 years	22 and 327	4.5	12	VIDAS in serum (bioMérieux)	645	10.1	95.4 (86.8; 98.9)	42.1 (38.1; 46.1)	0.11 (0.04; 0.33)
Lagares 2024 ≥65 years [29]	Adults ≥65 years	22 and 327	4.5	12	VIDAS in serum (bioMérieux)	793	14.38	100 (96.1; 100)	10.3 (8.2; 12.8)	0
Lapić 2024 [30]	Adults. Median age: 62. GCS 14–15.	35 and 400	2.5	12	Alinity i platform in plasma (Abbott)	62	11.3	100 (59.0; 100)	30.9 (19.1; 44.8)	0
Legramante 2024 [31]	Adults. Mean age: 54. Incl. GCS 13–15 (but all patients in the end had GCS 14–15)	35 and 400	2	12	Alinity i platform in serum (Abbott)	130	5.4	100 (59; 100)	27.6 (20.0; 36.4)	0
Li 2023 [32]	Adults. Mean age: 50.8. GCS 13–15 (72% GCS: 15)	22 and 327	1	6	ELISA in plasma/serum (Results are available in the article for serum and plasma. As these results are highly correlated, we report here merged results) (Banyan)	663	27	100 (98; 100)	13 (10; 16)	0
Oris 2024 (<3 h) [33]	Adults. Mean age: 59.1. Moderate risk within the mTBI according to French guidelines: GCS 14–15	30 and 360	1.7	3	i-STAT Alinity in plasma (Abbott)	192	9.8	100 (66.4; 100).	29 (22.5; 36.1)	0
Oris 2024 (<12 h) [33]	Adults. Mean age: 59.1. Moderate risk within the mTBI according to French guidelines: GCS 14–15	30 and 360	-	12	i-STAT Alinity in plasma (Abbott)	239	4.6	100 (73.5; 100)	31.7 (25.7; 38.2)	0
Oris 2024 (Oris 2024 [33] and Oris 2024 [34] share the same population. Oris 2024 [34] compares results with i-STAT Alinity and Alinity i platform. Only the latter is presented here as results with i-STAT Alinity is presented in Oris 2024 [33]) [34]	Adults. Mean age: 66.2. mTBI with intermediate risk as per French guidelines. GCS 13–15, (95.7% GCS 15)	35 and 400	1.7	12	Alinity i platform in plasma (Abbott)	230	4.8	100 (75.1; 100)	28.8 (22.9; 35.3)	0
Papa 2022 (cut-offs: GFAP 30, UCH-L1 327) [35]	Adults. Mean age 40. GCS 13–15 (90% GCSS 15).	30 and 327 (Two sets of prespecified biomarker cutoff values were used in the analysis based on previous studies. 30 ng/L GFAP and 327 ng/L UCH-L1 were the cut-offs used in the meta-analysis because they were the closest to cut-offs used in the assays for clinical use (i-STAT Alinity, Alinity i platform, VIDAS))	3	4	ELISA in serum (Banyan)	349	6.6	91 (70; 98)	20 (16; 24)	0.44 (0.12; 1.70) (A LR- greater than 1 means the test is worse than random guessing and behaves in the opposite way of a useful diagnostic, i.e., the probability of having a CT scan positive is greater after a negative test than before any test (probability pre-test))
Papa 2022 (cut-offs: GFAP 67, UCH-L1 189) [35]	Adults. Mean age 40. GCS 13–15 (90% GCSS 15).	67 and 189	3	4	ELISA in serum (Banyan)	349	6.6	100 (82; 100)	25 (20; 30)	0
Puravet 2025 [20]	Aged ≤16. Median age: 4.7 y. GCSS: 15 requiring hospitalisation or CT scan according to French guidelines	By age group (Cutt-off GFAP and UCH-L1 by age group <2 y 180 pg/mL and 373 pg/mL; 2–4 y 118 pg/mL and 272 pg/mL; >4 y 73 pg/mL and 217 pg/mL based on the 95th percentile for the mean of a control group of children. For S100B 0–9 months, greater than 0.35 μg/L; 10–24 months, greater than 0.23 μg/L; and older than 24 months, greater than 0.18 μg/L. The result combines the ages groups together)	2.4	3	Alinity i platform in serum (Abbott)	68	20.6	100 (75; 100)	42 (29; 56)	0
Trivedi 2024 (measured within 6 h) [36]	Adults. Median age: 47 GCS: 14–15 (88.7% GCS 15)	AUC	-	6	Simoa digital immunoassay in serum (Quanterix)	531	24.9	76 (66, 83)	83 (71, 90)	0.29 (0.22; 0.40)
Trivedi 2024 (measured within 6–9 h) [36]	NA (The characteristics of the patients are only available for the full sample)	AUC	-	6–9	Simoa digital immunoassay in serum (Quanterix)	248	47.6	80 (62, 86)	80 (60, 87)	0.25 (0.18; 0.37)
Trivedi 2024 (measured within 9–12 h) [36]	NA	AUC	-	9–12	Simoa digital immunoassay in serum (Quanterix)	154	43.5	73 (46, 82)	83 (57, 91)	0.32 (0.22; 0.49)
Welch 2025 [37]	Adults. Mean age: 49.1 GCS 13–15	35 and 400	3.2	12	Alinity i platform in plasma (Abbott)	1899	6.3	96.7 (91.7; 98.7)	40.1 (37.8; 42.4)	0.08 (0.03; 0.22)

**Table 2:** False negatives with lesions requiring neurosurgery.

Study ID	Proportion of False Negative Tests with Neurosurgically Manageable Lesions	Validated Head Rules
Bazarian 2018 [23]	0/3 false negative cases *	Not mentioned
Bazarian 2021 [24]	0/5 false negative cases	Not mentioned
Chayoua 2024 [25]	0/2 false negative cases	CT in Head Injury Patients (CHIP) decision rule
Curran 2025 [26]	0 false negative case	Not mentioned
Harris 2025 [27]	False negative cases not reported	Not mentioned
Iverson 2022 [21]	0 false negative case	Scandinavian guidelines
Kopcinovic 2025 [28]	?/5 false negative cases (diagnoses not reported)	Not mentioned
Ladang 2025 [22]	0/1 false negative case	Not mentioned
Lagares 2024 [29]	0/3 false negative cases **	Canadian CT Head Injury Rule (CCHR)
Lapić 2024 [30]	0 false negative case	Not mentioned
Legramante 2024 [31]	0 false negative case	Not mentioned
Li 2023 [32]	0 false negative case	Not mentioned
Oris 2024 [33]	0 false negative case	Société Française de Médecine d’Urgence (SFMU) criteria
Oris 2024 [34]	0 false negative case	Société Française de Médecine d’Urgence (SFMU) criteria
Papa 2022 [35]	0 false negative case with cut-offs GFAP 67 pg/mL, UCH-L1 189 pg/mL ?/2 false negative cases with cut-offs GFAP 30 pg/mL, UCH-L1 327 pg/mL (diagnoses not reported)	Not mentioned
Puravet 2025 [20]	0 false negative case	PECARN
Trivedi 2024 [36]	Not reported	Indication for CT scanning as determined by the physician in charge
Welch 2025 [37]	?/4 false negative cases (diagnoses not reported)	Not mentioned

*: 1 case might have been false CT positive (cavernous malformation); **: 2 cases might have been false CT positive (ruptured intracerebral aneurysm might have been the cause of the fall).

**Table 3:** Results of economic evaluations on GFAP and UCH-L1 in mTBI.

Study (Yr)	Costs	Outcomes	ICERs OR Alternative Summary Results	Sensitivity/Robustness
Su (2019) [39]	Not explicitly reported for each strategy.	CCTH only: 28.29 QALYs GFAP+UCH-L1: 28.29 QALYs. CCTH first, then GFAP+UCH-L1: 28.29 QALYs. GFAP+UCH-L1 first, then CCTH: 28.30 QALYs.	ICERs not calculated (missing cost for biomarker test). GFAP+UCH-L1 CE at WTP US$50,000, if cost/test ≤ US$308.96.	One-/two-/three-way. Monte Carlo simulation for effectiveness only. Overall robust results.
Zimmer (2023) [38]	GFAP+UCH-L1 vs. CT for all: -€4.15/patient CT scan: €568.43/patient. GFAP+UCH-L1: €564.28/patient. GFAP+UCH-L1 vs. S100β: -€4.73/patient S100β: €569.01/patient. GFAP+UCH-L1: €564.28/patient.	GFAP+UCH-L1 vs. CT for all: no diff. in QALYs/patient: GFAP+UCH-L1: 30.70. CT scan: 30.70. ↓ in CT scans: 29.7% (from 1096 to 771). GFAP+UCH-L1 vs. S100β: no diff in QALYs/patient: GFAP+UCH-L1: 30.70. S100β: 30.70. ↓ in CT scans: 5.6% (from 817 to 771).	GFAP+UCH-L1 less costly for equivalent * QALYs/patient.	One-way, scenarios, probabilistic; results robust for one way and scenarios. No details available on probabilistic.
Moya Torrecilla (2025) [40]	-€199/patient Standard care (CT): €899/patient. GFAP+UCH-L1: €700/patient.	Care time/patient: -111 min ↓ in patients with at least one CT scan: 28.5% (-927, from 3255 to 2328). ↓ in overall number of CT scans: 35.04% (-1464 from 4177 to 2713).	NA	Univariate SA: ±20%. Robust results, most sensitive to % positive biomarker and % of patients tested.

* Minimal differences in QALYs per patient in favour of the GFAP and UCH-L1 management strategy versus the CT scan for all (+0.00003 QALYs/patient) or the S100β (+0.00001 QALYs/patient); ↓ decrease.

## Data Availability

No new data were created or analysed in this study. This study is based on previously published data, which are cited within the article.

## Supplementary Materials

The following supporting information can be downloaded at: https://www.mdpi.com/article/10.3390/jcm15134858/s1: Supplement S1: Search strategies; Supplement S2: Inclusion and exclusion criteria for clinical studies (S2.1); Inclusion and exclusion criteria for economic studies (S2.2); Excluded clinical and costing studies after full text reading and reasons for exclusion (Table S2.3); Characteristics of included studies (Table S2.4); Meta-analysis performance of GFAP+UCH-L1 in adults, by analyser type (Figure S2.5); Ongoing studies on GFAP and UCH-L1 (Table S2.6); SROC curve of GFAP+UCH-L1 in adults (Figure S2.7); Comparative studies GFAP and UCH-L1 with S100β (Table S2.8). Deeks’ Funnel Plot (Figure S2.9).
