# Supplementary material for: GFAP and UCH-L1 for Ruling out Intracranial Lesions After Mild Traumatic Brain Injury: A Systematic Review and Meta-Analysis
Source: J Clin Med. 2026 Jun 23;15(13):4858. doi: 10.3390/jcm15134858 (PMC13361857; doi:10.3390/jcm15134858)
Supplement: Supplementary file 1 [file jcm-15-04858-s001.zip › Supplement S1_SearchStrategies.pdf]

SUPPLEMENT S1 - SEARCH STRATEGIES

Search strategies – Clinical Effectiveness Review

MEDLINE (OVID)

Database(s): Ovid MEDLINE(R) ALL May 22, 2025

Search Strategy:

#	Searches	Results
1	craniocerebral trauma/ or exp brain injuries/ or coma, post-head injury/ or exp head injuries, closed/ or head injuries, penetrating/ or exp intracranial hemorrhage, traumatic/ or exp skull fractures/	142845
2	((skull or cranial) adj3 fracture*).ti,ab.	5230
3	((head or brain or craniocerebral or cranial or cerebral or skull) adj4 (injur* or trauma*)).ti,ab.	156208
4	(trauma* and ((subdural or intracranial) adj2 (h?ematoma* or h?emorrhage* or bleed*))).ti,ab.	6826
5	or/1-4	221888
6	letter/	1295818
7	editorial/	726370
8	news/	230341
9	exp historical article/	415945
10	Anecdotes as Topic/	4747
11	comment/	1051116
12	case report/	0
13	(letter or comment*).ti.	213055
14	or/6-13	3011420
15	randomized controlled trial/ or random*.ti,ab.	1761907
16	14 not 15	2984004
17	animals/ not humans/	5305452
18	exp Animals, Laboratory/	998193
19	exp Animal Experimentation/	10709
20	exp Models, Animal/	682428
21	exp Rodentia/	3713855
22	(rat or rats or mouse or mice or rodent*).ti.	1529359
23	or/16-22	9326430

24 5 not 23170987

25 Biomarkers/ 385191

26 exp S100 Proteins/ 26824

27 Glial Fibrillary Acidic Protein/ 17518

28 Phosphopyruvate Hydratase/ 8758

29 Ubiquitin Thiolesterase/ 5957

30 MicroRNAs/ 141599

31 Brain-Derived Neurotrophic Factor/ 21419

32 Neurofilament Proteins/ 6813

33 Spectrin/ 3646

34 Myelin Basic Protein/ 7576

35 Extracellular Vesicles/ 15509

36 tau Proteins/ 21844

37 (Ubiquitin Thiolesterase* or "Ubiquitin C-terminal hydrolase*" or "Ubiquitin C-Terminal Esterase*" or "Ubiquitin Carboxy-Terminal Hydrolase*" or "Ubiquitin Carboxy-Terminal Esterase*" or uch-l1 or UCHL1).ti,ab. 2103

38 (S100* or GFAP or "glial fibrillary acid* protein*" or "brain-derived neurotrophic factor*" or "brain-derived nerve growth factor*" or BDNF or spectrin* or tau or proteomic* or microRNA* or miRNA* or micro-rna*).ti,ab. 429927

39 ((muscle or nervous or neuron* or alpha or beta or gamma) adj3 enolase*).ti,ab. 10244

40 (Phosphopyruvate Hydratase* or 2-phosphoglycerate* or 2-phospho-D-glycerate* or NSE).ti,ab. 8029

41 ((neurofilament* adj3 (protein* or chain* or polypeptide*)) or NF-L or NF-H).ti,ab. 8103

42 ((myelin basic or nerve tissue* or golli* or hog* or mbp*) adj2 protein*).ti,ab. 12397

43 (((extracellular or secretory) adj vesicle*) or exovesicle* or apoptotic bod* or exosome* or endosome* or ectosome* or microvesicle* or ((membrane or cell-derived) adj microparticle*)).ti,ab. 84463

44 biomarker*.ti,ab,kf. 514775

45 marker*.ti,ab. 1020958

46 or/25-45 2026425

47 randomized controlled trial.pt. 638639

48 controlled clinical trial.pt. 95680

49 randomi#ed.ti,ab. 897827

50 randomly.ti,ab. 461552

51 trial.ti. 336804

52 Meta-Analysis/ 216034

53 (meta analy* or metanaly* or metaanaly* or meta regression).ti,ab. 347834

54 ((systematic* or evidence*) adj3 (review* or overview*)).ti,ab. 466405

55 ((multiple treatment* or indirect or mixed) adj2 comparison*).ti,ab. 4424

56 Epidemiologic studies/ 9674

57 Observational study/ 175771

58 Cohort studies/ 357897

59 (cohort adj (study or studies or analys* or data)).ti,ab. 411900

60 ((follow up or observational or uncontrolled or non randomi#ed or epidemiologic*) adj (study or studies or data)).ti,ab. 430471

61 ((longitudinal or retrospective or prospective or cross sectional) and (study or studies or review or analys* or cohort* or data)).ti,ab. 2363183

62 Controlled Before-After Studies/ 782

63 Historically Controlled Study/ 238

64 Interrupted Time Series Analysis/ 2489

65 (before adj2 after adj2 (study or studies or data)).ti,ab. 11076

66 case control study/ 347919

67 case control*.ti,ab. 176331

68 Cross-sectional studies/ 544369

69 (cross sectional and (study or studies or review or analys* or cohort* or data)).ti,ab. 598121

70 or/47-69 4991303

71 Sensitivity.mp. and Specificity/ [mp=title, book title, abstract, original title, name of substance word, subject heading word, floating sub-heading word, keyword heading word, organism supplementary concept word, protocol supplementary concept word, rare disease supplementary concept word, unique identifier, synonyms, population supplementary concept word, anatomy supplementary concept word] 379582

72 (sensitivity or specificity).ti,ab. 1388938

73 ((pre test or pretest or post test) adj probability).ti,ab. 3683

74 (predictive value* or PPV or NPV).ti,ab. 166359

75 likelihood ratio*.ti,ab. 21815

76 Likelihood Functions/ 24328

77 ((area under adj4 curve) or AUC).ti,ab. 222454

78 (receive* operat* characteristic* or receive* operat* curve* or ROC curve*).ti,ab. 187149

79 gold standard.ab. 100907

80 exp Diagnostic Errors/ 124912

81 (false positiv* or false negativ*).ti,ab. 97932

82 Diagnosis, Differential/ 476806

83 (diagnos* adj3 (performance* or accurac* or utilit* or value* or efficien* or effectiveness or precision or validat* or validity or differential or error*)).ti,ab. 379751

84 or/71-83 2705034

85 24 and 46 and 70 and 84965

COCHRANE

Date: 26 May 2025

#1 MeSH Brain injuries, Traumatic 1798

#2 (brain OR head OR cerebral OR cortical) AND (concussion OR injury OR trauma) 18750

#3 #1 OR #2 18826

#4 #3 OR TBI 19643

#5 Biomarker OR marker 40918

#6 GFAP OR UCH 374

#7 Glial fibrillary acidic protein 228

#8 Ubiquitin 302

#9 #5 OR #6 OR #7 OR #8 41447

#10 minor OR mild OR minimal OR severity 264260

#11 sensitivity OR specificity OR "predictive value" OR predict*OR prognos*OR likelihood OR classification OR stratification OR diagnosis 341658

#12 #4 AND #9 AND #10 AND #11 208

EMBASE

Session Results 20 January 2025

No. Query Results Results

#34. #5 AND #30 AND #31 AND #32 AND [humans]/lim AND [embase]/lim AND [2022-2025]/py 624

#33. #5 AND #30 AND #31 AND #32 2,089

#32. #26 OR #27 OR #28 OR #29 2,629,213

#31. #15 OR #16 OR #17 OR #18 OR #19 OR #20 OR #21 OR #22 OR #23 OR #24 OR #25 7,267,954

#30. #6 OR #7 OR #8 OR #9 OR #10 OR #11 OR #12 OR #13 OR #14 2,706,950

#29. diagnos*:ti,ab AND (performance*:ti,ab OR accurac*:ti,ab OR utilit*:ti,ab OR value*:ti,ab OR efficien*:ti,ab OR effectiveness:ti,ab OR precision:ti,ab OR validat*:ti,ab OR validity:ti,ab OR differential:ti,ab OR error*:ti,ab)	1,568,428
#28. 'diagnostic accuracy' OR 'diagnostic test accuracy study' OR 'diagnostic error' OR ((false:ti,ab AND positiv*:ti,ab OR false:ti,ab) AND negativ*:ti,ab) OR 'differential diagnosis'	1,143,555
#27. ((receive*:ti,ab AND operat*:ti,ab AND characteristic*:ti,ab OR receive*:ti,ab) AND operat*:ti,ab AND curve*:ti,ab OR roc:ti,ab) AND curve*:ti,ab	237,056
#26. ('sensitivity' AND 'specificity' OR sensitivity:ti,ab OR specificity:ti,ab OR ((pre:ti,ab AND test:ti,ab OR pretest:ti,ab OR post:ti,ab) AND test:ti,ab AND probability:ti,ab) OR (predictive:ti,ab AND value*:ti,ab) OR ppv:ti,ab OR npv:ti,ab OR likelihood) AND ratio*:ti,ab OR (area:ti,ab AND under:ti,ab AND adj4:ti,ab AND curve:ti,ab) OR auc:ti,ab	523,406
#25. cross:ti,ab AND sectional:ti,ab AND (study:ti,ab OR studies:ti,ab OR review:ti,ab OR analys*:ti,ab OR cohort*:ti,ab OR data:ti,ab)	745,159
#24. 'cross-sectional study'	732,213
#23. case AND control*:ti,ab	689,392
#22. (longitudinal:ti,ab OR retrospective:ti,ab OR prospective:ti,ab OR cross:ti,ab) AND sectional:ti,ab AND (study:ti,ab OR studies:ti,ab OR review:ti,ab OR analys*:ti,ab OR cohort*:ti,ab OR data:ti,ab)	745,465
#21. ((follow:ti,ab AND up:ti,ab OR observational:ti,ab OR uncontrolled:ti,ab OR	623,264

non:ti,ab) AND randomized:ti,ab OR epidemiologic*:ti,ab) AND (study:ti,ab OR studies:ti,ab OR data:ti,ab)	
#20. cohort:ti,ab AND (study:ti,ab OR studies:ti,ab OR analys*:ti,ab OR data:ti,ab)	1,433,978
#19. cohort*:ti,ab	1,673,879
#18. (multiple:ti,ab AND treatment*:ti,ab OR indirect:ti,ab OR mixed:ti,ab) AND comparison*:ti,ab	103,116
#17. (systematic*:ti,ab OR evidence*:ti,ab) AND (review*:ti,ab OR overview*:ti,ab)	1,040,164
#16. (meta:ti,ab AND analy*:ti,ab OR metanaly*:ti,ab OR metaanaly*:ti,ab OR meta:ti,ab) AND regression:ti,ab	35,881
#15. random*:ti,ab OR factorial*:ti,ab OR ((crossover*:ti,ab OR cross:ti,ab) AND over*:ti,ab) OR ((doubl*:ti,ab OR singl*:ti,ab) AND blind*:ti,ab) OR assign*:ti,ab OR allocat*:ti,ab OR volunteer*:ti,ab OR placebo*:ti,ab OR 'crossover procedure' OR 'single blind procedure' OR 'randomized controlled trial' OR 'randomised controlled trial' OR 'double blind procedure' OR 'systematic review' OR 'meta-analysis'	4,097,043
#14. biomarker*:ti,ab,kw OR marker*:ti,ab	2,009,528
#13. ((extracellular:ti,ab OR secretory:ti,ab) AND vesicle*:ti,ab OR exovesicle*:ti,ab OR apoptotic:ti,ab) AND bod*:ti,ab OR exosome*:ti,ab OR endosome*:ti,ab OR ectosome*:ti,ab OR microvesicle*:ti,ab OR ((membrane:ti,ab OR 'cell derived':ti,ab) AND microparticle*:ti,ab)	90,341
#12. ((myelin:ti,ab AND basic:ti,ab OR nerve:ti,ab) AND tissue*:ti,ab OR golli*:ti,ab OR hog*:ti,ab	27,296

OR mbp*:ti,ab) AND protein*:ti,ab	
#11. neurofilament*:ti,ab AND adj3:ti,ab AND (protein*:ti,ab OR chain*:ti,ab OR polypeptide*:ti,ab) OR 'nf l':ti,ab OR 'nf h':ti,ab	1,680
#10. phosphopyruvate:ti,ab AND hydratase*:ti,ab OR '2 phosphoglycerate*':ti,ab OR '2 phospho d glycerate*':ti,ab OR nse:ti,ab	10,969
#9. (muscle:ti,ab OR nervous:ti,ab OR neuron*:ti,ab OR alpha:ti,ab OR beta:ti,ab OR gamma:ti,ab) AND enolase*:ti,ab	12,381
#8. s100*:ti,ab OR gfap:ti,ab OR 'glial fibrillary acid* protein*':ti,ab OR 'brain-derived neurotrophic factor*':ti,ab OR 'brain-derived nerve growth factor*':ti,ab OR bdnf:ti,ab OR spectrin*:ti,ab OR tau:ti,ab OR proteomic*:ti,ab OR microrna*:ti,ab OR mirna*:ti,ab OR 'micro rna*':ti,ab	547,295
#7. ubiquitin:ti,ab AND thiolesterase*:ti,ab OR 'ubiquitin c-terminal hydrolase*':ti,ab OR 'ubiquitin c-terminal esterase*':ti,ab OR 'ubiquitin carboxy-terminal hydrolase*':ti,ab OR 'ubiquitin carboxy-terminal esterase*':ti,ab OR 'uch l1':ti,ab OR uchl1:ti,ab	3,089
#6. 'biological marker'/exp OR 'protein s 100' OR 'glial fibrillary acidic protein' OR 'enolase' OR 'ubiquitin thiolesterase' OR 'microrna' OR 'brain derived neurotrophic factor' OR 'neurofilament protein' OR 'spectrin' OR 'myelin basic protein' OR 'exosome' OR 'tau protein'	996,359
#5. #1 OR #2 OR #3 OR #4	544,345
#4. 'head injury'/exp OR 'brain injury'/exp OR 'skull injury'/exp OR 'skull fracture'/exp	389,356

#3. trauma*:ti,ab AND (subdural:ti,ab OR intracranial:ti,ab) AND (h?ematoma*:ti,ab OR h?emorrhage*:ti,ab OR bleed*:ti,ab)	4,637
#2. (skull:ti,ab OR cranial:ti,ab) AND fracture*:ti,ab	11,484
#1. (head:ti,ab OR brain:ti,ab OR craniocerebral:ti,ab OR cranial:ti,ab OR cerebral:ti,ab OR skull:ti,ab) AND (injur*:ti,ab OR trauma*:ti,ab)	318,023

EMBASE Update May 2025

Session Results

No. Query Results	Results
#41. #38 AND #39 AND [2025-2025]/py	437
#40. #38 AND #39	3,850
#39. #20 AND #21 AND #22	8,599
#38. #23 OR #24 OR #25 OR #26 OR #27 OR #28 OR #29 OR #30 OR #31 OR #32 OR #33 OR #34 OR #35 OR #36 OR #37	8,354,823
#37. cross:ti,ab AND sectional:ti,ab AND (study:ti,ab OR studies:ti,ab OR review:ti,ab OR analys*:ti,ab OR cohort*:ti,ab OR data:ti,ab)	745,445
#36. 'cross-sectional study'	732,524
#35. case AND control*:ti,ab	689,498
#34. (longitudinal:ti,ab OR retrospective:ti,ab OR prospective:ti,ab OR cross:ti,ab) AND sectional:ti,ab AND (study:ti,ab OR studies:ti,ab OR review:ti,ab OR analys*:ti,ab OR cohort*:ti,ab OR data:ti,ab)	745,751
#33. (follow:ti,ab AND up:ti,ab OR observational:ti,ab OR uncontrolled:ti,ab OR ((non:ti,ab AND randomized:ti,ab OR non:ti,ab) AND	2,409,440

randomized:ti,ab) OR epidemiologic*:ti,ab) AND (study:ti,ab OR studies:ti,ab OR data:ti,ab)	
#32. cohort:ti,ab AND (study:ti,ab OR studies:ti,ab OR analys*:ti,ab OR data:ti,ab)	1,434,348
#31. cohort*:ti,ab	1,674,304
#30. (multiple:ti,ab AND treatment*:ti,ab OR indirect:ti,ab OR mixed:ti,ab) AND comparison*:ti,ab	103,137
#29. (systematic*:ti,ab OR evidence*:ti,ab) AND (review*:ti,ab OR overview*:ti,ab)	1,040,507
#28. (meta:ti,ab AND analy*:ti,ab OR metanaly*:ti,ab OR metaanaly*:ti,ab OR meta:ti,ab) AND regression:ti,ab	35,895
#27. 'crossover procedure' OR 'single blind procedure' OR 'randomized controlled trial' OR 'randomised controlled trial' OR 'double blind procedure' OR 'systematic review' OR 'meta-analysis'	1,882,700
#26. assign*:ti,ab OR allocat*:ti,ab OR volunteer*:ti,ab OR placebo*:ti,ab	1,360,230
#25. (doubl*:ti,ab OR singl*:ti,ab) AND blind*:ti,ab	324,873
#24. (crossover*:ti,ab OR cross:ti,ab) AND over*:ti,ab	428,387
#23. random*:ti,ab OR factorial*:ti,ab	2,196,411
#22. #9 OR #10 OR #11 OR #12 OR #13 OR #14 OR #15 OR #16 OR #17 OR #18 OR #19	4,199,301
#21. #5 OR #6 OR #7 OR #8	2,275,897
#20. #1 OR #2 OR #3 OR #4	568,019
#19. diagnos*:ti,ab AND (performance*:ti,ab OR accurac*:ti,ab OR utilit*:ti,ab OR value*:ti,ab OR efficien*:ti,ab OR effectiveness:ti,ab OR precision:ti,ab OR validat*:ti,ab OR validity:ti,ab OR differential:ti,ab OR error*:ti,ab)	1,568,794
#18. 'differential diagnosis'	513,952

#17. (false:ti,ab AND positiv*:ti,ab OR false:ti,ab) AND negativ*:ti,ab	82,227
#16. 'diagnostic accuracy' OR 'diagnostic test accuracy study' OR 'diagnostic error'	609,710
#15. ((receive*:ti,ab AND operat*:ti,ab AND characteristic*:ti,ab OR receive*:ti,ab) AND operat*:ti,ab AND curve*:ti,ab OR roc:ti,ab) AND curve*:ti,ab	237,147
#14. area:ti,ab AND under:ti,ab AND adj4:ti,ab AND curve:ti,ab OR auc:ti,ab	239,910
#13. likelihood AND ratio*:ti,ab	69,953
#12. predictive:ti,ab AND value*:ti,ab OR ppv:ti,ab OR npv:ti,ab	338,574
#11. (pre:ti,ab AND test:ti,ab OR pretest:ti,ab OR post:ti,ab) AND test:ti,ab AND probability:ti,ab	10,780
#10. sensitivity:ti,ab OR specificity:ti,ab	1,744,349
#9. 'sensitivity' AND 'specificity'	713,392
#8. gfap:ti,ab OR 'glial fibrillary acid* protein*':ti,ab	40,538
#7. ubiquitin:ti,ab AND thiolesterase*:ti,ab OR 'ubiquitin c-terminal hydrolase*':ti,ab OR 'ubiquitin c-terminal esterase*':ti,ab OR 'ubiquitin carboxy-terminal hydrolase*':ti,ab OR 'ubiquitin carboxy-terminal esterase*':ti,ab OR 'uch l1':ti,ab OR uchl1:ti,ab	3,089
#6. 'glial fibrillary acidic protein' OR 'ubiquitin thiolesterase'	45,916
#5. 'biological marker'/exp OR biomarker*:ti,ab,kw OR marker*:ti,ab	2,093,739
#4. 'head injury'/exp OR 'brain injury'/exp OR 'skull injury'/exp OR 'skull fracture'/exp	389,412
#3. trauma*:ti,ab AND (subdural:ti,ab OR intracranial:ti,ab) AND (h?ematoma*:ti,ab OR	4,638

h?emorrhage*:ti,ab OR bleed*:ti,ab)

#2.	(skull:ti,ab OR cranial:ti,ab) AND fracture*:ti,ab	11,485
#1.	(head:ti,ab OR brain:ti,ab OR craniocerebral:ti,ab OR cranial:ti,ab OR cerebral:ti,ab OR skull:ti,ab) AND (injur*:ti,ab OR trauma*:ti,ab)	318,069

Search strategies – Cost-Effectiveness Review

MEDLINE

Database(s): **Ovid MEDLINE(R) ALL** 1946 to July 14, 2025

N.	Searches	Results
1	craniocerebral trauma/ or exp brain injuries/ or coma, post-head injury/ or exp head injuries, closed/ or head injuries, penetrating/ or exp intracranial hemorrhage, traumatic/ or exp skull fractures/	143487
2	((skull or cranial) adj3 fracture*).ti,ab.	5271
3	((head or brain or craniocerebral or cranial or cerebral or skull) adj4 (injur* or trauma*)).ti,ab.	157679
4	(trauma* and ((subdural or intracranial) adj2 (h?ematoma* or h?emorrhage* or bleed*))).ti,ab.	6896
5	Biomarkers/	389031
6	Glial Fibrillary Acidic Protein/	17586
7	Ubiquitin Thiolesterase/	6031
8	(Ubiquitin Thiolesterase* or "Ubiquitin C-terminal hydrolase*" or "Ubiquitin C-Terminal Esterase*" or "Ubiquitin Carboxy-Terminal Hydrolase*" or "Ubiquitin Carboxy-Terminal Esterase*" or uch-l1 or UCHL1).ti,ab.	2132
9	(S100* or GFAP or "glial fibrillary acid* protein*" or "brain-derived neurotrophic factor*" or "brain-derived nerve growth factor*" or BDNF or spectrin* or tau or proteomic* or microRNA* or miRNA* or micro-rna*).ti,ab.	435429
10	biomarker*.ti,ab,kf.	526107
11	marker*.ti,ab.	1031967
12	Sensitivity.mp. and Specificity/ [mp=title, book title, abstract, original title, name of substance word, subject heading word, floating sub-heading word, keyword heading word, organism supplementary concept word, protocol supplementary concept	380944

	word, rare disease supplementary concept word, unique identifier, synonyms, population supplementary concept word, anatomy supplementary concept word]	
13	(sensitivity or specificity).ti,ab.	1403814
14	((pre test or pretest or post test) adj probability).ti,ab.	3712
15	(predictive value* or PPV or NPV).ti,ab.	168378
16	likelihood ratio*.ti,ab.	22045
17	Likelihood Functions/	24381
18	((area under adj4 curve) or AUC).ti,ab.	227838
19	(receive* operat* characteristic* or receive* operat* curve* or ROC curve*).ti,ab.	191159
20	exp Diagnostic Errors/	125180
21	(false positiv* or false negativ*).ti,ab.	98766
22	Diagnosis, Differential/	477764
23	(diagnos* adj3 (performance* or accurac* or utilit* or value* or efficien* or effectiveness or precision or validat* or validity or differential or error*)).ti,ab.	385622
24	1 or 2 or 3 or 4	223541
25	5 or 6 or 7 or 8 or 9 or 10 or 11	1935304
26	12 or 13 or 14 or 15 or 16 or 17 or 18 or 19 or 20 or 21 or 22 or 23	2665452
27	24 and 25 and 26	2309
28	exp Economics/	758061
29	exp Health Care Costs/	75337
30	(cost and cost analysis).mp.	60847
31	exp Economics, Pharmaceutical/ or exp Economics, Medical/ or exp Economics, Hospital/ or exp Economics, Nursing/	47160
32	exp "Value of Life"/	5838
33	exp Cost-Benefit Analysis/	98340
34	(cost effectiveness or cost-effectiveness).mp.	89715
35	(cost utility or cost-utility).mp.	7371
36	exp Quality-Adjusted Life Years/	18101
37	exp Health Expenditures/	27723
38	budget*.mp.	47078
39	(price or prices or pricing).mp.	59399

40	exp "Costs and Cost Analysis"/	280426
41	28 or 29 or 30 or 31 or 32 or 33 or 34 or 35 or 36 or 37 or 38 or 39 or 40	870285
42	27 and 41	10

EMBASE

Embase session results (15 Jul 2025)

No. Query Results

#38 #20 AND #21 AND #22 AND #37 100

#37 #23 OR #24 OR #25 OR #26 OR #27 OR #28 OR #29 OR #30 OR #31 OR #32 OR #33 OR #34 OR #35 OR #36 1002561

#36 budget* AND [embase]/lim 53664

#35 'budget impact analysis' AND [embase]/lim 2861

#34 (price OR prices OR pricing) AND [embase]/lim105052

#33 'cost minimization analysis'/exp AND [embase]/lim 4225

#32 'cost utility analysis'/exp AND [embase]/lim 13768

#31 'hospital cost'/exp AND [embase]/lim 40746

#30 'cost control'/exp AND [embase]/lim 60098

#29 'cost of illness'/exp AND [embase]/lim 12586

#28 'cost effectiveness analysis'/exp AND [embase]/lim 203055

#27 'cost benefit analysis'/exp AND [embase]/lim 71392

#26 'health care financing'/exp AND [embase]/lim 13556

#25 'health economics'/exp AND [embase]/lim 886912

#24 'health care cost'/exp AND [embase]/lim 326777

#23 'economics'/exp AND [embase]/lim 18876

#22 #9 OR #10 OR #11 OR #12 OR #13 OR #14 OR #15 OR #16 OR #17 OR #18 OR #194199301

#21 #5 OR #6 OR #7 OR #8 2275897

#20 #1 OR #2 OR #3 OR #4 568019

#19 diagnos*:ti,ab AND (performance*:ti,ab OR accurac*:ti,ab OR utilit*:ti,ab OR value*:ti,ab OR efficien*:ti,ab OR effectiveness:ti,ab OR precision:ti,ab OR validat*:ti,ab OR validity:ti,ab OR differential:ti,ab OR error*:ti,ab)1568794

#18 'differential diagnosis' 513952

#17 (false:ti,ab AND positiv*:ti,ab OR false:ti,ab) AND negativ*:ti,ab82227

#16 'diagnostic accuracy' OR 'diagnostic test accuracy study' OR 'diagnostic error' 609710

#15 ((receive*:ti,ab AND operat*:ti,ab AND characteristic*:ti,ab OR receive*:ti,ab) AND operat*:ti,ab AND curve*:ti,ab OR roc:ti,ab) AND curve*:ti,ab 237147

#14 area:ti,ab AND under:ti,ab AND adj4:ti,ab AND curve:ti,ab OR auc:ti,ab 239910

#13 likelihood AND ratio*:ti,ab 69953

#12 predictive:ti,ab AND value*:ti,ab OR ppv:ti,ab OR npv:ti,ab 338574

#11 (pre:ti,ab AND test:ti,ab OR pretest:ti,ab OR post:ti,ab) AND test:ti,ab AND probability:ti,ab 10780

#10 sensitivity:ti,ab OR specificity:ti,ab 1744349

#9 'sensitivity' AND 'specificity' 713392

#8 gfap:ti,ab OR 'glial fibrillary acid* protein*':ti,ab 40538

#7 ubiquitin:ti,ab AND thiolesterase*:ti,ab OR 'ubiquitin c-terminal hydrolase*':ti,ab OR 'ubiquitin c-terminal esterase*':ti,ab OR 'ubiquitin carboxy-terminal hydrolase*':ti,ab OR 'ubiquitin carboxy-terminal esterase*':ti,ab OR 'uch l1':ti,ab OR uchl1:ti,ab 3089

#6 'glial fibrillary acidic protein' OR 'ubiquitin thiolesterase' 45916

#5 'biological marker'/exp OR biomarker*:ti,ab,kw OR marker*:ti,ab 2093739

#4 'head injury'/exp OR 'brain injury'/exp OR 'skull injury'/exp OR 'skull fracture'/exp 389412

#3 trauma*:ti,ab AND (subdural:ti,ab OR intracranial:ti,ab) AND (h?ematoma*:ti,ab OR h?emorrhage*:ti,ab OR bleed*:ti,ab) 4638

#2 (skull:ti,ab OR cranial:ti,ab) AND fracture*:ti,ab 11485

#1 (head:ti,ab OR brain:ti,ab OR craniocerebral:ti,ab OR cranial:ti,ab OR cerebral:ti,ab OR skull:ti,ab) AND (injur*:ti,ab OR trauma*:ti,ab) 318069

ECONLIT

19	5 and 10 and 18	0
18	11 or 12 or 13 or 14 or 15 or 16 or 17	511
17	UCH-L1.mp.	0
16	Ubiquitine carboxyl-terminal hydrolase L1.mp.	0
15	GFAP.mp.	0
14	Glial Fibrillary Acidic Protein.mp.	0
13	biomarker.mp.	107
12	marker.mp.	394
11	(biological adj marker*).mp.	19
10	6 or 7 or 8 or 9	2198
9	(brain adj concussion).mp.	0
8	traumatic.mp.	295
7	trauma.mp.	375
6	injury.mp.	1604
5	1 or 2 or 3 or 4	6250
4	cerebral.mp.	39

3	cortical.mp.	30
2	brain.mp.	1841
1	head.mp.	4387
