# Supplementary material for: GFAP and UCH-L1 for Ruling out Intracranial Lesions After Mild Traumatic Brain Injury: A Systematic Review and Meta-Analysis
Source: J Clin Med. 2026 Jun 23;15(13):4858. doi: 10.3390/jcm15134858 (PMC13361857; doi:10.3390/jcm15134858)
Supplement: Supplementary file 1 [file jcm-15-04858-s001.zip › Supplement S2_Methods_additional_Tablesfigures.pdf]

SUPPLEMENT S2 – GFAP IN COMBINATION WITH UCH- L1 IN MILD TRAUMATIC BRAIN INJURIES

S 2.1. Inclusion and exclusion criteria for clinical studies:

A systematic review was performed to assess the performance of GFAP and UCH-L1 used in combination to exclude ICIs in adults and children with mTBI, CT scan or magnetic resonance imaging (MRI) being considered the gold standard (reference test). The protocol of our systematic review was registered at CRD420251051158. We followed the reporting recommendations of the PRISMA-DTA framework.
The following databases were searched in January 2025, and updated in May 2025: MEDLINE via OVID, Embase, and Cochrane Database of Systematic Reviews. The sites of other HTA agencies were visited to identify any recent assessments in this field and the references of any relevant studies were checked. A full search strategy is available in Supplement 1.

Any original study meeting the following criteria was considered **eligible**:
<u>Population:</u>

- Any patient (child, adult and elderly) presenting to the ED with a mTBI who is eligible for a CT scan or MRI according to the current diagnostic criteria.

<u>Index test:</u>

- GFAP and UCH-L1 in combination.

<u>Reference standard:</u>

- CT scan or MRI.

<u>Target conditions to be excluded</u>:

- Any form of intracranial brain injury detected by a CT scan or MRI, including conditions requiring urgent neurosurgery (epidural or subdural haematoma, subarachnoid haemorrhage).

<u>Main outcomes</u>:

- Sensitivity (Se) and specificity (Sp), and negative likelihood ratio (LR-).

<u>Study types</u>:

- Primary diagnosis studies. Literature reviews were only used for reference checking purposes, and only primary studies were included in our review.

<u>**Exclusion** criteria</u>:

- Studies on the clinical prognosis of mTBI.
- Studies limited to patients with severe head injuries or mixed populations for which results for patients with mTBI could not be isolated.
- Studies on patients with no injuries (e.g. degenerative disease).
- Studies reporting only the area under the curve (AUC) of the Receiver Operator Curve (ROC), without mentioning specific cut-offs for computation of sensitivity and specificity. These studies were excluded because they do not fit the aim of our research, i.e. assessing if measuring biomarkers allows to rule out a brain injury.

Study selection was performed by two researchers, while data extraction was performed by one researcher and cross-checked by a second one. Study selection was done in two steps, first on title and

abstract, and second on content. Excel was used in order to capture information on a standardized way on both the characteristics of the studies and their relevant outcomes. The following information was extracted from each included article: study design; number and characteristics of patients (age, GCS score, incidence of brain lesions confirmed by CT scans, exclusion factors); assay techniques; cut-offs for GFAP and UCH-L1; time interval between trauma and assay; number of test which were true positive, false positive, true negative, and false negative relative to CT results.

S 2.2. Inclusion and exclusion criteria for economic studies:

Any original economic evaluation meeting the following criteria was considered **eligible**:

Population:

- Patients presenting at the ED with mTBI (i.e. GCS 13-15) following a trauma accident.

Index test:

- GFAP and UCH-L1 in combination.

Reference standard:

- CT scan or MRI.

Design:

Analyses comparing GFAP and UCH-L1 versus head CT from both a cost and an accuracy perspective.

Type of publication:

- Primary studies or reviews.

The following **exclusion criteria** were applied:

Population:

- Moderate or severe TBIs.

Index test:

- Any biomarkers other than GFAP and UCH-L1, or studies evaluating these biomarkers individually.

Reference standard:

- Clinical practice not considering CT scans or MRI.

Design:

- Pricing comparisons, or cost descriptions no taking into consideration test accuracy.

Type of publication:

- Letters, editorials, notes, abstracts, posters

Table S 2.3 – Excluded clinical and costing studies after full text reading and reasons for exclusion

Study author and year	Reasons for exclusion
	**CLINICAL LITERATURE**
**Anderson 2020**[1]	Population: moderate and severe cases
**Biberthaler 2021**[2]	Population: mild and moderate cases mixed
**Bielanin 2024**[3]	Narrative review
**Bohmer 2011**[4]	Severe and moderate cases
**Cevik 2019**[5]	Sample mixing children and adults
**Chen 2022**[6]	Population: mild and moderate cases mixed
**Chiollaz 2024**[7]	Intervention studied
**Czeiter 2020**[8]	Population: mild and moderate cases mixed
**Datwyler 2023**[9]	Abstract
**Diaz-Arrastia 2014**[10]	Population: mild and moderate cases mixed
**Forouzan 2021**[11]	Intervention studied
**Gardner 2018**[12]	Intervention studied
**Hellewell 2020**[13]	No appropriate reference standard
**Keski-Pukkila 2024**[14]	Intervention studied
**Korley 2022**[15]	Population: mild and moderate cases mixed and outcome wrong
**Ladang 2024**[16]	Abstract
**Mahan 2019**[17]	Population: mild and moderate cases mixed
**Malhotra Armaan 2022**[18]	Abstract
**Mastandrea 2025**[19]	Abstract
**McMahon 2015**[20]	Population: mild and moderate cases mixed
**Middleton 2022**[21]	Study design
**Morell-Garcia 2023**[22]	Abstract
**Munoz Pareja 2024**[23]	Population children: mild and moderate cases mixed
**Polat 2022**[24]	Intervention studied
**Okonkwo 2013**[25]	Population: mild and moderate cases mixed
**Okonkwo 2020**[26]	Intervention studied
**Papa 2012**[27]	Population: mild and moderate cases mixed
**Papa 2012**[28]	Population: mild and moderate cases mixed
**Papa 2014**[29]	Only individuals presenting with extracranial fractures considered
**Papa 2022**[30]	No data to calculate accuracy
**Papa 2023**[31]	Population: mild and moderate cases mixed
**Pecorano 2024**[32]	Abstract
**Posti 2019**[33]	Intervention studied
**Puccio 2022**[34]	Abstract
**Puccio 2024**[35]	No data to calculate accuracy

Study author and year	Reasons for exclusion
**CLINICAL LITERATURE**	
**Ryan 2022**[36]	Population: No CT scan recommended in most patients
**Santos Marzano 2022**[37]	Intervention studied
**Tsitsipanis 2023**[38]	Population: mild and moderate cases mixed
**Wang 2023**[39]	Population: mild and moderate cases mixed
**Welch 2016**[40]	Population: mild and moderate cases mixed
**Whitehouse 2025**[41]	Intervention studied
**ECONOMIC LITERATURE**	
**Calluy 2024**[42]	Abstract
**Gregorio 2025**[43]	Abstract
**Menacho Roman 2024**[44]	Study Design: Review of studies
**Zimmer 2020**[45]	Abstract

Table S 1.4– Characteristics of included studies

Study ID	Country	Design	Population - Inclusion	Glasgow Coma Scale	Exclusion criteria	Outcome on CT
Bazarian 2018[46]	22 sites: 15 USA; Canada, 7 Europe<br>Dec 2012-March 2014	Secondary analysis of banked samples (samples from the ALERT-TBI study)	Adults (mean age 49). LoC or amnesia not required for inclusion. Non-contrast head CT scanning within 12h of injury.	14-15 (14=92, 15=1828)	Time of injury could not be determined, head CT scanning not performed, venepuncture not feasible, or informed consent not obtainable.	CT-positive: acute epidural haematoma, acute subdural haematoma, indeterminate extraaxial haemorrhage, intraventricular haemorrhage, parenchymal haematoma, petechial haemorrhagic or bland sheer injury, subarachnoid haemorrhage, brain oedema, brain herniation, non-haemorrhagic contusion, ventricular compression, ventricular trapping, cranial fractures, depressed skull fractures, facial fractures, scalp injury, or skull base fractures
Bazarian 2021[47]	22 sites: 15 USA; Canada, 7 Europe<br>Dec 2012-March 2014	Secondary analysis of banked samples (samples from the ALERT-TBI study)	Adults (mean age 49) mTBI GCS 13–15. LoC or amnesia not required for inclusion. Non-contrast head CT scanning, <12h	13-15 (13 n=22, 14 n=90, 15 n=1789)	Time of injury could not be determined, head CT scanning not performed, venepuncture not feasible, or informed consent not obtainable.	CT-positive: acute epidural hematoma, acute subdural hematoma, intraventricular hemorrhage, parenchymal hemorrhage/contusion, petechial hemorrhage/bland sheer injury, subarachnoid hemorrhage, brain

Study ID	Country	Design	Population - Inclusion	Glasgow Coma Scale	Exclusion criteria	Outcome on CT
						edema/herniation, and ventricular compression/trapping.
Chayoua 2024[48]	Netherlands: ED in two level 1 trauma centers<br>Jan 2020-Dec 2022	Cross-sectional study with consecutive enrollment	Adults (median age 48) mTBI: GCSS 13-15 and LOC ≤30 min and/or amnaesia ≤24 hrs. CT scan decided based on CHIP-decision rule[49]	13-15	Significant neurologic or psychiatric comorbidity, prior hospital admission for TBI, drug abuse, mental disability, language barriers, or illiteracy	CT scans were scored according to the Marshall score. Scores were dichotomized as negative (Marshall score 1, no abnormalities) or positive (Marshall score >1, indicating lesions, compressed cisterns, or midline shifts).
Curran 2025[50]	Tertiary referral trauma centre in Scotland	Cross-sectional study of unclear design (not stated if consecutive enrollment was applied)	Adult patients ≥18 years (mean age 63, alcohol intox 34%), <12 hours head injury, CT head scan was requested	13-15	Penetrating head injury; poly-trauma; blood sampling >12 hours from injury; insufficient sample volume, and lack of test calibration materials	CT+ defined as: any traumatic intra or extra-axial haemorrhage or shear-type injury or traumatic brain oedema +/- associated herniation or ventriculomegaly
Harris 2024[51]	18 U.S. Level I trauma ED<br>Feb 2014 - Aug 2018	Cross-sectional retrospective analysis on banked samples from the TRACK-TBI study (nonconsecutive enrollment)	≥17, presented to ED, clinically-indicated non-contrast brain CT within 24 h of TBI.<br>Acute TBI as defined by the American Congress of Rehabilitation Medicine (ACRM) Criteria: sustained traumatically induced physiological disruption of brain function, as manifested by more than one of the following: any period of loss of consciousness, any loss of memory for events (e.g., amnesia) immediately before or after the accident,	Results in suppl for GCS 13-15 in patients without blood alcohol level available (not obtained clinically)	Prisoner or in police custody, pregnant, psychiatric hold, major debilitating baseline mental health disorders, or major debilitating neurological disease; participants in an interventional trial; penetrating head injury; or spinal cord injury with an American Spinal Injury Association (ASIA) score of C or worse	CT+ defined as the presence of any intracranial lesion as defined by the National Institute of Neurological Disorders and Stroke (NINDS) common data elements (CDEs) for neuroimaging.[52] For a list of lesions see Iverson. [53]

Study ID	Country	Design	Population - Inclusion	Glasgow Coma Scale	Exclusion criteria	Outcome on CT
			any alteration of mental state at the time of the accident (feeling dazed, disoriented, and/or confused), and any focal neurologic deficits that may or may not be permanent.			
Iverson 2022[53]	Finland 1 ED neurosurgical referral centre<br>Level 1 trauma<br>Nov 2015 and Nov 2016	Cross-sectional retrospective analysis on banked samples	Older patients ≥60 (mean age 79), acute TBI within 24hrs. Either blunt injury to the head or acceleration/deceleration type injury resulting in witnessed LoC, disorientation, or amnesia	14-15	Seen after 24hrs from injury<br>GCS<14	NINDS CDEs: skull fracture, epidural hematoma, subdural hematoma, subarachnoid hemorrhage, vascular dissection, traumatic aneurysm, venous sinus injury midline shift, cisternal compression, fourth ventricle shift/effacement, contusion, intracerebral hemorrhage, intraventricular hemorrhage, diffuse axonal injury, penetrating injuries, craniocervical junction injury, brain swelling, ischemia/infarction/hypoxic-ischemic injury. Excluded: isolated skull fracture
Kopcinovic 2025[54]	12 healthcare centers from 6	Cross-sectional study; consecutive enrollment unclear	Adult patients ≥18 years (median age: 64; 49%>65y) with suspected mTBI caused by external mechanical force, attendance to the ED within 12 h from a head injury,	13-15	Unknown time of injury, head CT scan not performed, and inability to obtain a blood sample. Demographics, medical history, GCS, alcohol	A positive head CT was defined as the presence of acute intracranial hemorrhage or hematoma

Study ID	Country	Design	Population - Inclusion	Glasgow Coma Scale	Exclusion criteria	Outcome on CT
	European countries (Croatia, Poland, Portugal, Romania, Slovak Republic, and Slovenia)<br><br>Feb 2022-June 2024		a GCS 13–15 assessed at the time of admission, and a head CT performed within 12 h of head trauma. Suspected mTBI was assessed and clinically evaluated as per locally instituted routine protocols or guidelines		consumption, injury mechanism, and CT results were collected.	
Ladang 2025[55]	Greece (corresponding author from BE) ED general hospital Athens<br><br>2022-2023	Cross-sectional study with consecutive enrollment	Adults (median age 63) with CT scan decided by neurosurgeon. Indication of brain CT scan: neurological focal deficit; anterograde amnesia; GCS <15 after 2 h post-TBI; suspicion of vault depression fracture; fracture of the basal skull; persisting nausea, vomiting or headache; post-TBI seizures; pre-injury treatment with antithrombotic drugs; loss of consciousness or amnesia with age >65 years, fall >1m or hit pedestrian. 68.8 % CT-; 31.2 % CT+.	13-15	Children; GCS 3–12; 1ary admission non-traumatic neurological disorder; diagnosed neurodegenerative disease or other neurological disorder; blood collection not feasible, undetermined time of injury; penetrating head trauma; on mechanical ventilation; 1ary diagnosis of ischemic or hemorrhagic stroke or transient ischemic attack last 6 months or history of neurosurgery procedure in last 90 days	Positive or negative result not defined.<br><br>(Experienced radiologist with Philips ingenuity 5,000 CT scanner)
Lagares 2024[56, 57]	France: 11 trauma centres and 1 community hospital; Spain 3 trauma	Cross-sectional analysis of data at baseline from BRAINI study. Not clear if enrollment was consecutive. Total follow-up 3 months.	≥18 France, ≥15 Spain: (median age 63; range 16-100)<br><br>TBI: alteration in brain function<br><br>CT scan performed based on Canadian Head Rule and other risk factors i.e neurological focal deficit; anterograde amnesia; GCS <15 after 2h post TBI;	13-15<br><br>(13 n=9 or 1%)	GCS 3–12, unknown time of injury, time of CT scan or blood sampling unknown or >12h after TBI, admission for a non-traumatic neurological disorder, penetrating head injury, neuropsychiatric and neurological comorbidities that might interfere with the evaluation, venipuncture not	A CT-positive: (1) epidural haematoma, (2) acute subdural haematoma, (3) subarachnoid haemorrhage, (4) intraventricular haemorrhage, (5) intraparenchymal

Study ID	Country	Design	Population - Inclusion	Glasgow Coma Scale	Exclusion criteria	Outcome on CT
	centres and 1 one community hospital<br>August 2019-June 2021		suspicion of vault depression fracture; fracture of the basal skull; persisting nausea, vomiting or headache; post-TBI seizures; preinjury treatment with antithrombotic drugs; loss of consciousness or amnesia in patients over 65 years of age; fall more than 1m or hit pedestrian; and any other condition requiring a CT according to the in-charge physician		feasible, brain CT scan not performed, pregnant or breastfeeding	contusion, (6) petechial haemorrhage or (7) any finding related to diffuse axonal injury and depressed skull fracture. Linear skull fractures were recorded but were not included in the definition of a CT-positive result.
Lapić 2024[58]	Croatia tertiary hospital ED – level 1 trauma centre<br>May 2022-March 2023	Cross-sectional study; no consecutive enrollment	Adult were selected for mild neurological symptoms: headache, vertigo, nausea, vomiting, alterations in mental status such as confusion, amnesia, LoC <30 min, disorientation.<br>CT decided by ED physician after examination<br>Those with acute head trauma (median age: 62); <12h were then included. Patients with a neurological diagnosis excluded.	14-15	>12h after head trauma, neurological or psychiatric disorders, neurosurgical interventions, and/or previous traumatic brain lesions confirmed by CT<br>Patients with presenting symptoms attributed to gastrointestinal disease, sepsis, pneumonia, non-neurological disorder were not included.	Intracranial abnormality (results section); not further specified
Legramante 2024[59]	Italy 1 centre<br>Oct 2022-Jan 2023	Cross-sectional retrospective study on banked samples	>18 year (mean age: 54) with mild cranial trauma and head CT scan<br>Within 12h	13-15<br>(0% 13)	GCS score < 13; CRP >5mg/L; anticoagulant therapy; venous sampling >12h after trauma	Positive result in the clinical notes; not further defined
Li 2023[60]	USA one centre with 24h neurosurgery facilities	Cross-sectional retrospective study of ED data and banked samples; non-	Adults (mean age: 50.8) with suspected mTBI brought to the centre by ambulance or helicopter. All had a CT scan.	13-15 (72% 15)	Adults in which a CT scan or a blood sample was not performed. Non-English–speaking patients and patients without the capacity to consent	Presence/absence of closed head injuries including skull fracture, pneumocephalus, hemorrhage, mass effect,

Study ID	Country	Design	Population - Inclusion	Glasgow Coma Scale	Exclusion criteria	Outcome on CT
	specialised TBI<br>Dec 2015-Apr 2017	consecutive (Monday – Friday)				and brain parenchymal injuries. The volumes of each type of hematoma or contusion, as well as the extent of midline shift, were quantified as continuous variables, while the extent of subarachnoid hemorrhage, intraventricular hemorrhage, brain edema/swelling, cisternal compression, and hydrocephalus was characterised on ordinal scales.
Oris 2024[61]	France 1 ED<br>Jan 2023-June 2023	Cross-sectional retrospective analysis on banked samples (except for S100ß); consecutive enrollment	Adult (mean age 59.1); at least one associated risk factor: antiplatelet monotherapy, loss of consciousness or posttraumatic amnesia of facts 30 min before the injury. Patients with S100ß levels above the decision threshold of 0.10 µg/L (<3h) underwent CT scanning. Sampling within 12h. For the other patients (blood sampling >3 h), a CT scan was systematically performed.<br>Since 2022, the new French guidelines also recommend the blood determination of "GFAP and UCH-L1" for the management of mTBI patients with blood sampling <12h.	14-15	Children. Low or high risk within the mTBI according to the French guidelines: GCSS ≤13	CT scans were considered positive if any signs of cranial (skull fracture) or intracranial pathology (hematoma, air, or contusion) were present.

Study ID	Country	Design	Population - Inclusion	Glasgow Coma Scale	Exclusion criteria	Outcome on CT
Oris 2024[62]	France 1 ED<br>Jan 2023-June 2023	Cross-sectional retrospective analysis on banked samples (except for S100ß); consecutive enrollment	Adult (mean age 66.2) mTBI patients intermediate risk as per the French guidelines: routine S100ß Cobas® system (Roche Diagnostics, Meylan, France) for patients who underwent blood sampling within 3h of mTBI. Patients with S100ß levels above the decision threshold of 0.10 µg/L underwent CT scanning. Sampling within 12h. Patients>3h no S100ß	13-15 (95.7% 15)	mTBI patients defined as having a low or high risk as per the French guidelines.	CT scans were considered positive if any signs of cranial (skull fracture) or intracranial pathology (hematoma, air, or contusion) were present.
Papa 2022[63]	USA ED level I trauma center<br>March 2010-March 2014	Cross-sectional study based on a convenience sample	Adults (mean age 40); blunt head trauma followed by a change in sensorium such as LoC, amnesia, or disorientation. mTBI determined by physician; within 4h injury; most patients with blunt head injury and subsequent symptoms have a head CT scan performed as part of usual care.	GCS 13-15 (90% 15).	<18years; no history of trauma as their 1ary event; known dementia, chronic psychosis, or active central nervous system pathology; pregnant; incarcerated; or systolic blood pressure < 100mmHg	Acute traumatic intracranial lesion CT scan, including extra-axial lesions (eg, epidural, subdural, and subarachnoid hemorrhage) or intra-axial lesions (eg, contusions, intraparenchymal hemorrhage, cerebral edema, traumatic axonal injury, and midline shift of intracranial contents), as well as any signs of brain herniation or pneumocephalus.
Puravet 2025[64]	France; ED of 11 hospitals.<br>Nov 2016-Oct 2021	Cross-sectional retrospective analysis on banked samples from a S100ß stepped wedge cluster randomised trial	Children≤16 (median age 4.7) GCS 15 requiring hospitalisation or CT scan according French Pediatric Society guidelines (PECARN-based). Within 3h of injury.<br>< 2 GCS paediatric, inclusion if parietal or occipital scalp haematoma, a loss of	15	Enrolment in another therapeutic trial involving drug administration; having Down syndrome or melanoma; the trauma occurring more than 3 h before admission to hospital; being in the group at high risk as per the PECARN	Cranial CT result: Positive for intracranial brain injury e.g. Epidural haematoma, Haemorrhagic contusion, Subdural haematoma, Pneumocephalus,

Study ID	Country	Design	Population - Inclusion	Glasgow Coma Scale	Exclusion criteria	Outcome on CT
			consciousness for more than 5s, serious accident, atypical behaviour according to the parents. ≥2 inclusion if loss of consciousness for any length of time, vomiting, serious accident, severe headache		algorithm; having mTBI that did not require hospitalisation or a CT scan	Subarachnoid haemorrhage, Othematoma
Trivedi 2024[65]	65 sites (includes UZA) across 18 countries Dec 2014-Dec 2017; Trauma Level 1=60, Level 2=4, Level 3=1	Retrospective analysis CENTER-TBI study of banked samples; convenience sample	TBI adults with GCS 14-15 on admission. Clinical suspicion of TBI, indication for CT scanning as determined by physician in charge and presentation to a study center within 24h of injury.	14-15 (score 15 within 6h 88.7%)	Patients with severe pre-existing neurological disorders. Patients with GCS ≤13, with missing data on CT scanning, and/or aged under 18	Clinically significant traumatic CT abnormality (mass lesion, extra-axial hemorrhage, epidural hemorrhage [EDH], acute or chronic subdural hemorrhage [SDH], contusion, traumatic axonal injury, traumatic subarachnoid hemorrhage, intraventricular hemorrhage, midline shift, or cisternal compression). A skull fracture was included as a CT abnormality only if it was a depressed fracture or if the subject was taking antithrombotic medications, in accordance with NICE guidance.
Welch 2025[66]	USA 15 sites, 7 in Europe	Retrospective analysis of data and banked blood samples ALERT-TBI trial;	Adults (mean age 49); ED or other healthcare facility with a non-penetrating head injury; GSC 13-15; banked blood available	13-15 (94.1% score 15)	Patients for whom injury time could not be determined, blood samples could not be obtained within 6h, a CT	any sign of study-defined traumatic intracranial injury: acute epidural hematoma, acute subdural

Study ID	Country	Design	Population - Inclusion	Glasgow Coma Scale	Exclusion criteria	Outcome on CT
	Dec 2012 to March 2014	nonconsecutive enrollment	CT scan as part of routine care, evaluation by the treating physician.		scan was not deemed necessary, or appropriate consent was not obtained.	hematoma, intraventricular hemorrhage, parenchymal hemorrhage/contusion, petechial hemorrhage/bland sheer injury, subarachnoid hemorrhage, brain edema/herniation, or ventricular compression/trapping. Skull fractures without any of the above intracranial findings were not considered positive

Figure S 2.5 – Meta-analysis performance of GFAP+UCH-L1 in adults, by analyser type (n=10 440)

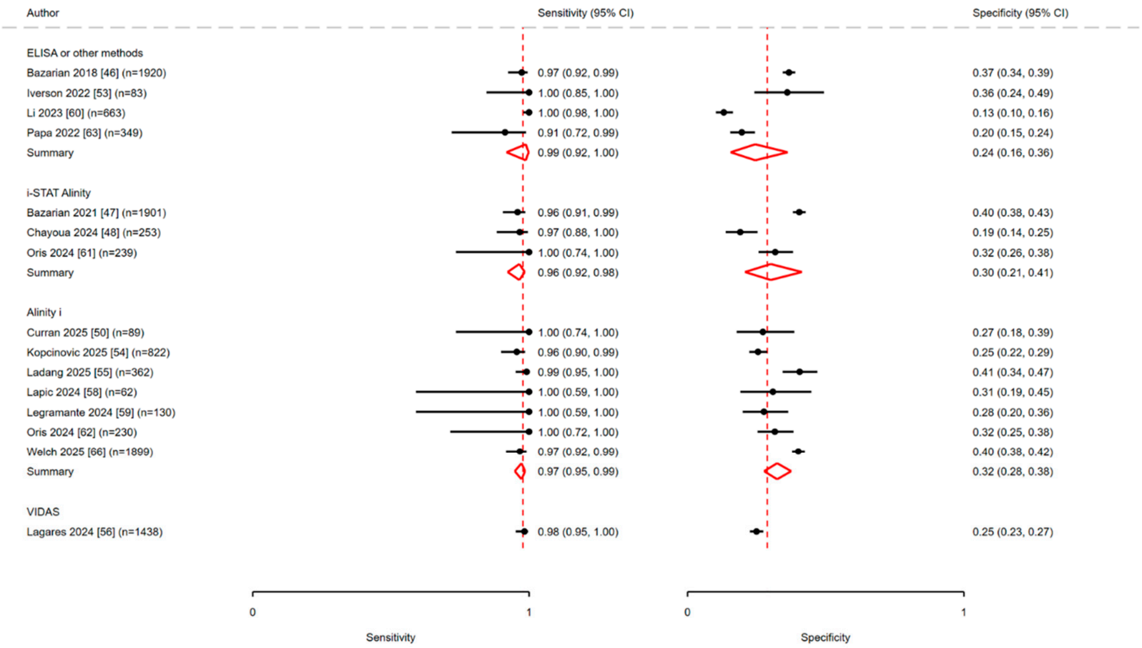

Table S 2.6 – On-going studies on GFAP and UCH-L1 referenced in ClinicalTrials.gov (30/11/2025)

NCT	Title	Population	Completion date
NCT05425251	BRAINI-2 Elderly MTBI European Study67	OLDER_ADULT	Mar-25
NCT05413499	Blood Biomarkers to Improve Management of Children With TBI (BRAINI-2) 68	CHILD	Apr-25
NCT05108909	Rapid Diagnosis and Prognosis Recognition of Imaging and Biomarkers in Mild to Moderate TBI	ADULT, OLDER_ADULT	Aug-25
NCT06766435	Evaluation of the Abbott i-STAT TBI Biomarker Test	ADULT, OLDER_ADULT	Oct-25
NCT06449183	VIDAS® TBI Real Life Performance in Subjects with Mild TBI (mTBI)	ADULT, OLDER_ADULT	Nov-25
NCT05885529	Glial Fibrillary Acidic Protein (GFAP) and Ubiquitin Carboxy-terminal Hydrolase L1 (UCH-L1) to Exclude Lesions Linked to Significant Traumatic Brain Injuries	ADULT, OLDER_ADULT	Mar-26
NCT06940232	Validating a Blood Test for the Detection of TBI in Children	CHILD	Dec-27
NCT05588115	Rapid, Accurate, Cost-effective Assessment of Blood Biomarkers for Diagnosis of Concussion	ADULT, OLDER_ADULT	Dec-27
NCT05964764	Validation of the Scandinavian Guidelines for Minor and Moderate Head Trauma in Children	CHILD	Dec-29
NCT04602806	Transforming Research and Clinical Knowledge in TBI (TRACK-TBI) Precision Medicine Phase 2 Option 1	ADULT, OLDER_ADULT	Apr-30

Figure S 2.7 – SROC curve of GFAP+UCH-L1 in adults

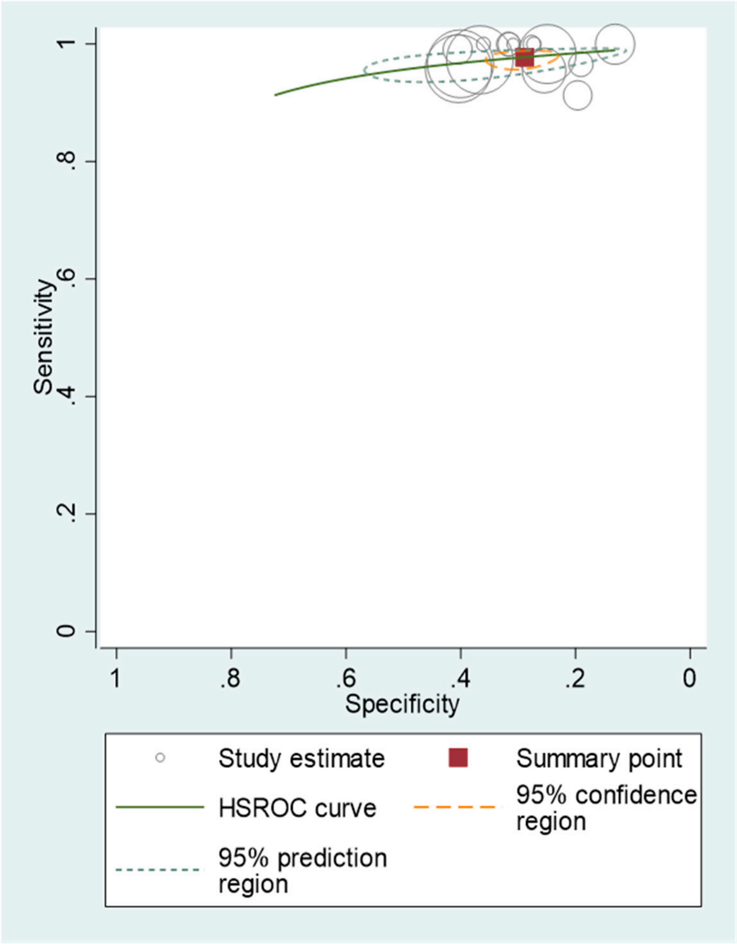

Table S2.8 – Comparative studies GFAP and UCH-L1 with S100β

Study ID		Biomarker	Cut-off values	Measured within (hrs)	Analyser (provider)	N	CT+ %	Sensitivity % (95% CI)	Specificity % (95% CI)	LR-
Lagares 2024	*Adults in FR and ≥15 in Sp. Median age 69. GCSS 13–15*	*S100β*	*0.1 µg/L*	*6*	*Electrochemiluminescence immunoassay (ECLIA) in serum (Cobas®, Roche)*	*846*	*11.7*	*87.8 (79.8; 93.6)*	*34.8 (31.4; 38.3)*	*0.35 (0.20; 0.60)*
		GFAP+UCH-L1	*GFA: 22 pg/ml + UCH-L1: 327 pg/ml*		*VIDAS in serum (bioMérieux)*	*1438*	*12.2*	*98.3 (95; 99.7)*	*24.9 (22.6; 27.4)*	*0.07 (0.02; 0.21)*
Li 2023	*Adults. Mean age: 50.8. GCS 13-15. (72% GCS: 15)*	*S100β*	*105 pg/mL*	*6*	*Electrochemiluminescence immunoassay (ECLIA) in serum (Cobas®, Roche)*	*189*	*23.3*	*93.2 (81.3; 98.6)*	*17.2 (11.5; 24.4)*	*0.40 (0.13; 1.25)*
		GFAP+UCH-L1	*22pg/ml (GFAP) and 327pg/ml (UCH-L1)*	*6*	*ELISA in plasma/serum 1 (Banyan)*	*663*	*27*	*100 (98; 100)*	*13 (10; 16)*	*0*
Oris 2024	*Adults. Mean age: 59.1. Moderate risk within the mTBI according to French guidelines: GCS 14-15*	*S100β*	*0.1 µg/L*	*3*	*Electrochemiluminescence immunoassay (ECLIA) in serum (Cobas®, Roche)*	*192*	*9.8*	*100 (66.4; 100)*	*25.7 (19.5; 32.6)*	*0*
		GFAP+UCH-L1	*30pg/ml (GFAP) and 360pg/ml (UCH-L1)*	*3*	*i-STAT Alinity in plasma (Abbott)*	*192*	*9.8*	*100 (66.4; 100).*	*29 (22.5; 36.1)*	*0*
				12		*239*	*4.6*	*100 (73.5; 100)*	*31.7 (25.7; 38.2)*	*0*
Puravet 2025	*Aged ≤16. Median age: 4.67. GCSS:*	*S100β*		*3*	*Electrochemiluminescence immunoassay*	*68*	*20.6*	*92 (64; 100)*	*51.9 (37.8; 65.7)*	*0.15 (0.02; 1.01)*

1 Results are available in the article for serum and plasma. As these results are highly correlated, we report here merged results.

Study ID		Bio marker	Cut-off values	Measured within (hrs)	Analyser (provider)	N	CT + %	Sensitivity % (95% CI)	Specificity % (95% CI)	LR-
	15 requiring hospitalisation or CT scan according to French guidelines		*By age group2*		*(ECLIA) in serum* *(Cobas®, Roche)*					
		GFAP+UCH-L1		*3*	*Alinity i platform in serum (Abbott)*	*68*	*20.6*	*100 (75; 100)*	*42 (29; 56)*	*0*
Trivedi 2024	*Adults. Median age 47 (ICI present), 58 (no ICI present) GCS 14-15 (88.7% GCS 15).*	*S100 β*	*AUC*	*6*	*Electrochemiluminescence immunoassay (ECLIA) in serum* *(Cobas®, Roche)*	*531*	*24.9*	*78 (68; 83)*	*61 (56; 66)*	*0.36 (0.26; 0.50)*
		GFAP+UCH-L1	*AUC*	*6*	*Simoa digital immunoassay in serum (Quanterix)*	*531*	*24.9*	*76 (66, 83)*	*83 (71, 90)*	*0.29 (0.22; 0.40)*

2 Cutt-off GFAP and UCH-L1 by age group <2y 180 pg/mL and 373 pg/mL; 2-4y 118 pg/mL and 272 pg/mL; >4y 73 pg/mL and 217 pg/mL based on the 95th percentile for the mean of a control group of children. For S100β 0–9 months, greater than 0·35 µg/L; 10–24 months, greater than 0·23 µg/L; and older than 24 months, greater than 0·18 µg/L. The result combines the ages groups together.

Figure S 2.9 – Deeks' Funnel Plot

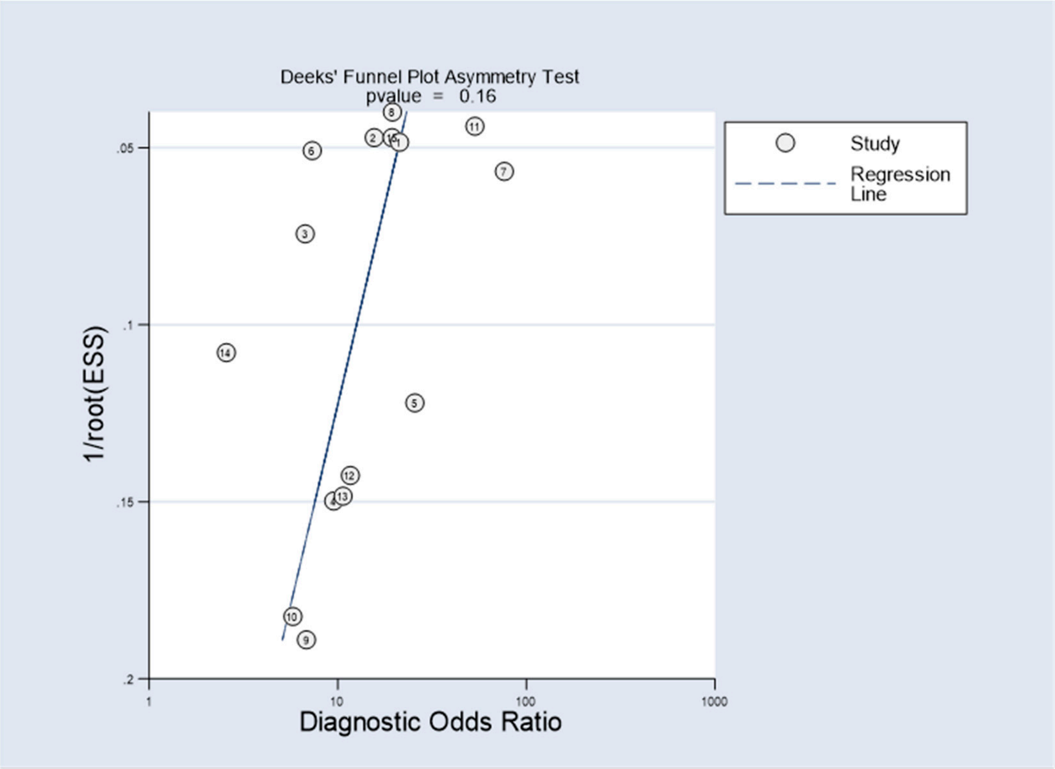

REFERENCES

1. Anderson TN, Hwang J, Munar M, Papa L, Hinson HE, Vaughan A, et al. Blood-based biomarkers for prediction of intracranial hemorrhage and outcome in patients with moderate or severe traumatic brain injury. J Trauma Acute Care Surg. 2020;89(1):80-6.
2. Biberthaler P, Musaelyan K, Krieg S, Meyer B, Stimmer H, Zapf J, et al. Evaluation of Acute Glial Fibrillary Acidic Protein and Ubiquitin C-Terminal Hydrolase-L1 Plasma Levels in Traumatic Brain Injury Patients with and without Intracranial Lesions. Neurotrauma Rep. 2021;2(1):617-25.
3. Bielanin JP, Metwally SAH, Paruchuri SS, Sun D. An overview of mild traumatic brain injuries and emerging therapeutic targets. Neurochem Int. 2024;172:105655.
4. Böhmer AE, Oses JP, Schmidt AP, Perón CS, Krebs CL, Oppitz PP, et al. Neuron-specific enolase, S100B, and glial fibrillary acidic protein levels as outcome predictors in patients with severe traumatic brain injury. Neurosurgery. 2011;68(6):1624-30; discussion 30-1.
5. Çevik S, Özgenç MM, Güneyk A, Evran Ş, Akkaya E, Çalış F, et al. NRGN, S100B and GFAP levels are significantly increased in patients with structural lesions resulting from mild traumatic brain injuries. Clin Neurol Neurosurg. 2019;183:105380.
6. Chen H, Ding VY, Zhu G, Jiang B, Li Y, Boothroyd D, et al. Association between Blood and Computed Tomographic Imaging Biomarkers in a Cohort of Mild Traumatic Brain Injury Patients. J Neurotrauma. 2022;39(19-20):1329-38.
7. Chiollaz AC, Pouillard V, Spigariol F, Romano F, Seiler M, Ritter Schenk C, et al. Management of Pediatric Mild Traumatic Brain Injury Patients: S100b, Glial Fibrillary Acidic

Protein, and Heart Fatty-Acid-Binding Protein Promising Biomarkers. Neurotrauma Rep. 2024;5(1):529-39.

8. Czeiter E, Amrein K, Gravesteijn BY, Lecky F, Menon DK, Mondello S, et al. Blood biomarkers on admission in acute traumatic brain injury: Relations to severity, CT findings and care path in the CENTER-TBI study. EBioMedicine. 2020;56:102785.
9. Datwyler S, R. Chandran, J.A. Marino, D.R. West, H.N. Syed, Z. Al Sahouri, et al. A-094 Performance Evaluation of GFAP and UCH-L1 Biomarkers for Traumatic Brain Injury in the Alinity i TBI Test (in development). Clinical Chemistry 69:S1 133. 2023.
10. Diaz-Arrastia R, Wang KK, Papa L, Sorani MD, Yue JK, Puccio AM, et al. Acute biomarkers of traumatic brain injury: relationship between plasma levels of ubiquitin C-terminal hydrolase-L1 and glial fibrillary acidic protein. J Neurotrauma. 2014;31(1):19-25.
11. Forouzan A, Barzegari H, Hosseini O, Delirrooyfard A. The Diagnostic Competence of Glial Fibrillary Acidic Protein in Mild Traumatic Brain Injury and Its Prognostic Value in Patient Recovery. Turk Neurosurg. 2021;31(3):355-60.
12. Gardner RC, Rubenstein R, Wang KKW, Korley FK, Yue JK, Yuh EL, et al. Age-Related Differences in Diagnostic Accuracy of Plasma Glial Fibrillary Acidic Protein and Tau for Identifying Acute Intracranial Trauma on Computed Tomography: A TRACK-TBI Study. J Neurotrauma. 2018;35(20):2341-50.
13. Hellewell SC, Conquest A, Little L, Vallance S, Board J, Bellomo R, et al. EPO treatment does not alter acute serum profiles of GFAP and S100B after TBI: A brief report on the Australian EPO-TBI clinical trial. J Clin Neurosci. 2020;76:5-8.
14. Keski-Pukkila M, Karr JE, Posti JP, Berghem K, Kotilainen AK, Blennow K, et al. Preliminary Evaluation of the Scandinavian Guidelines for Initial Management of Minimal, Mild, and Moderate Head Injuries with Glial Fibrillary Acidic Protein. Neurotrauma Rep. 2024;5(1):50-60.
15. Korley FK, Jain S, Sun X, Puccio AM, Yue JK, Gardner RC, et al. Prognostic value of day-of-injury plasma GFAP and UCH-L1 concentrations for predicting functional recovery after traumatic brain injury in patients from the US TRACK-TBI cohort: an observational cohort study. Lancet Neurol. 2022;21(9):803-13.
16. Ladang A, I. Trifonidi, G. Vavoulis, C. Leens, A. Perot, L. Vranken, et al. C6 MILD TRAUMATIC BRAIN INJURIES CAN BE EFFECTIVELY RULED-OUT BY MTBI TEST FROM ABBOTT BUT TEST SPECIFICITY CAN BE INCREASED BY AGED-DEPENDENT CUT-OFFS. Clin Chem Lab Med 2024; 62(3): A1–A46. 2024.
17. Mahan MY, Thorpe M, Ahmadi A, Abdallah T, Casey H, Sturtevant D, et al. Glial Fibrillary Acidic Protein (GFAP) Outperforms S100 Calcium-Binding Protein B (S100B) and Ubiquitin C-Terminal Hydrolase L1 (UCH-L1) as Predictor for Positive Computed Tomography of the Head in Trauma Subjects. World Neurosurg. 2019;128:e434-e44.
18. Malhotra Armaan K, Ide Kentaro, Salaheen Zaid, Mahood Quenby, Guerguerian Anne-Marie, Hutchison Jamie. CCN-RCC Abstracts: Biomarkers for Diagnosis and Prognostication of Mild Traumatic Brain Injury in Children: A Systematic Review. Journal of Head Trauma Rehabilitation. 2022;page E412.
19. Mastandrea P, Mengozzi S, Bernardini S. Systematic review and meta-analysis of observational studies evaluating glial fibrillary acidic protein (GFAP) and ubiquitin C-terminal hydrolase L1 (UCHL1) as blood biomarkers of mild acute traumatic brain injury (mTBI) or sport-related concussion (SRC) in adult subjects. Diagnosis (Berl). 2025;12(1):1-16.
20. McMahon PJ, Panczykowski DM, Yue JK, Puccio AM, Inoue T, Sorani MD, et al. Measurement of the glial fibrillary acidic protein and its breakdown products GFAP-BDP biomarker for the detection of traumatic brain injury compared to computed tomography and magnetic resonance imaging. J Neurotrauma. 2015;32(8):527-33.
21. Middleton J. UCH-L1 and GFAP Testing (i-STAT TBI Plasma) for the Detection of Intracranial Injury Following Mild Traumatic Brain Injury. Am Fam Physician. 2022;105(3):313-4.

22. Morell-Garcia D, J. Ortega , Viles M, M. Santes Berto , A. Ballesteros , M. Marin, et al. Poster 2038 IMPLEMENTATION OF NEW SERUM BIOMARKERS OF ACUTE BRAIN INJURY: DIAGNOSTIC-THERAPEUTIC IMPLICATIONS IN EMERGENCY DEPARTMENT. Clin Chem Lab Med 61, suppl pp S87. 2023.
23. Munoz Pareja JC, de Rivero Vaccari JP, Chavez MM, Kerrigan M, Pringle C, Guthrie K, et al. Prognostic and Diagnostic Utility of Serum Biomarkers in Pediatric Traumatic Brain Injury. J Neurotrauma. 2024;41(1-2):106-22.
24. Polat ZM, Yucel M, Cikriklar HI, Altındiş M, Yurumez Y. Investigation of Early Diagnostic Value of Glial Fibrillary Acidic Protein and Ubiquitin C-Terminal Hydrolase Blood Levels in Minor Head Trauma in Turkey. Clin Lab. 2022;68(8).
25. Okonkwo DO, Yue JK, Puccio AM, Panczykowski DM, Inoue T, McMahon PJ, et al. GFAP-BDP as an acute diagnostic marker in traumatic brain injury: results from the prospective transforming research and clinical knowledge in traumatic brain injury study. J Neurotrauma. 2013;30(17):1490-7.
26. Okonkwo DO, Puffer RC, Puccio AM, Yuh EL, Yue JK, Diaz-Arrastia R, et al. Point-of-Care Platform Blood Biomarker Testing of Glial Fibrillary Acidic Protein versus S100 Calcium-Binding Protein B for Prediction of Traumatic Brain Injuries: A Transforming Research and Clinical Knowledge in Traumatic Brain Injury Study. J Neurotrauma. 2020;37(23):2460-7.
27. Papa L, Lewis LM, Falk JL, Zhang Z, Silvestri S, Giordano P, et al. Elevated levels of serum glial fibrillary acidic protein breakdown products in mild and moderate traumatic brain injury are associated with intracranial lesions and neurosurgical intervention. Ann Emerg Med. 2012;59(6):471-83.
28. Papa L, Lewis LM, Silvestri S, Falk JL, Giordano P, Brophy GM, et al. Serum levels of ubiquitin C-terminal hydrolase distinguish mild traumatic brain injury from trauma controls and are elevated in mild and moderate traumatic brain injury patients with intracranial lesions and neurosurgical intervention. J Trauma Acute Care Surg. 2012;72(5):1335-44.
29. Papa L, Silvestri S, Brophy GM, Giordano P, Falk JL, Braga CF, et al. GFAP out-performs S100β in detecting traumatic intracranial lesions on computed tomography in trauma patients with mild traumatic brain injury and those with extracranial lesions. J Neurotrauma. 2014;31(22):1815-22.
30. Papa L, Rosenthal K, Cook L, Caire M, Thundiyil JG, Ladde JG, et al. Concussion severity and functional outcome using biomarkers in children and youth involved in organized sports, recreational activities and non-sport related incidents. Brain Inj. 2022;36(8):939-47.
31. Papa L, Brophy GM, Alvarez W, Hirschl R, Cress M, Weber K, et al. Sex differences in time course and diagnostic accuracy of GFAP and UCH-L1 in trauma patients with mild traumatic brain injury. Sci Rep. 2023;13(1):11833.
32. Pecoraro V, G. Micali, M. Cuccorese, M. Surgo, M. Ravazzini, T. Trenti. P0376 The accuracy of serum GFAP, UCH-L1 AND S100 for excluding mild traumatic brain injury in emergency room. Clinica Chimica Acta 558 (2024) 118821. 2024.
33. Posti JP, Takala RSK, Lagerstedt L, Dickens AM, Hossain I, Mohammadian M, et al. Correlation of Blood Biomarkers and Biomarker Panels with Traumatic Findings on Computed Tomography after Traumatic Brain Injury. J Neurotrauma. 2019;36(14):2178-89.
34. Puccio A, Frederick Korley, David Okonkwo, Ramon Diaz-Arrastia, Ester Yuh, John Yue, et al. P309 DIAGNOSTIC UTILITY OF GFAP BEYOND 24 HOURS OF ACUTE TRAUMATIC BRAIN INJURY: A TRACK-TBI STUDY. JOURNAL OF NEUROTRAUMA 39:A-1–A-128. 2022.
35. Puccio AM, Yue JK, Korley FK, Okonkwo DO, Diaz-Arrastia R, Yuh EL, et al. Diagnostic Utility of Glial Fibrillary Acidic Protein Beyond 12 Hours After Traumatic Brain Injury: A TRACK-TBI Study. J Neurotrauma. 2024;41(11-12):1353-63.

36. Ryan E, Kelly L, Stacey C, Duff E, Huggard D, Leonard A, et al. Traumatic Brain Injury in Children: Glial fibrillary Acidic Protein and Clinical Outcomes. Pediatr Emerg Care. 2022;38(3):e1139-e42.
37. Marzano LAS, Batista JPT, de Abreu Arruda M, de Freitas Cardoso MG, de Barros J, Moreira JM, et al. Traumatic brain injury biomarkers in pediatric patients: a systematic review. Neurosurg Rev. 2022;45(1):167-97.
38. Tsitsipanis C, Miliaraki M, Paflioti E, Lazarioti S, Moustakis N, Ntotsikas K, et al. Inflammation biomarkers IL-6 and IL-10 may improve the diagnostic and prognostic accuracy of currently authorized traumatic brain injury tools. Exp Ther Med. 2023;26(2):364.
39. Wang KK, Munoz-Pareja JC, Lautenslager LA, Tyndall JA, Yang Z, Kerrigan MR, et al. Diagnostic performance of point-of-care ubiquitin carboxy-terminal Hydrolase-L1 assay in distinguishing imaging abnormalities in traumatic brain injury: A TRACK-TBI cohort study. Advances in Biomarker Sciences and Technology. 2023;5:38-49.
40. Welch RD, Ayaz SI, Lewis LM, Unden J, Chen JY, Mika VH, et al. Ability of Serum Glial Fibrillary Acidic Protein, Ubiquitin C-Terminal Hydrolase-L1, and S100B To Differentiate Normal and Abnormal Head Computed Tomography Findings in Patients with Suspected Mild or Moderate Traumatic Brain Injury. J Neurotrauma. 2016;33(2):203-14.
41. Whitehouse D, Mikolić A, Czeiter E, Richter S, Buki A, Wang KK, et al. Serum Biomarkers as Adjuncts to the National Institute for Health and Care Excellence Head Injury Guidelines (NG232, 2023) When Selecting Patients with Traumatic Brain Injury for Computed Tomography: A Collaborative European NeuroTrauma Effectiveness Research in Traumatic Brain Injury Study. J Neurotrauma. 2025.
42. Calluy E, Cavalier, E., & Ladang, A. . Study of the cost-effectiveness of the mTBI test, the Canadian CT Head Rule and the CT scan in an adult population. Annual meeting of the Royal Belgian Society of Laboratory Medicine., Brussels, Belgium. 2024.
43. Gregorio DJ, Yu H, Delp K, Kupas DF. 29 Utility of Bioassay by Age Group to Detect Intracranial Hemorrhage in Adults with Mild Traumatic Brain Injury. JACEP Open. 2025;6(2).
44. Menacho Román M, Penedo Alonso JR, Morales Rodríguez A, Pecharromán de Las Heras I, Vicente Bartulos A, Arribas Gómez I, et al. Hospital-based health technology assessment of a screening rapid test MTBI (GFAP and UCH-L1 blood biomarkers) for mild traumatic brain injury. Int J Technol Assess Health Care. 2024;41(1):e5.
45. Zimmer L, McDade C, Purser M, Blanc E, Pavlov V, Earnshaw S. The cost-effectiveness of blood-based brain biomarkers for screening adults with mild traumatic brain injury H. Beyhaghi1. Intensive Care Medicine Experimental. 2020;8(SUPPL 2).
46. Bazarian JJ, Biberthaler P, Welch RD, Lewis LM, Barzo P, Bogner-Flatz V, et al. Serum GFAP and UCH-L1 for prediction of absence of intracranial injuries on head CT (ALERT-TBI): a multicentre observational study. Lancet Neurol. 2018;17(9):782-9.
47. Bazarian JJ, Welch RD, Caudle K, Jeffrey CA, Chen JY, Chandran R, et al. Accuracy of a rapid glial fibrillary acidic protein/ubiquitin carboxyl-terminal hydrolase L1 test for the prediction of intracranial injuries on head computed tomography after mild traumatic brain injury. Acad Emerg Med. 2021;28(11):1308-17.
48. Chayoua W, Visser K, de Koning ME, Beishuizen A, R IJ, van der Naalt J, et al. Evaluation of Glial Fibrillary Acidic Protein and Ubiquitin C-Terminal Hydrolase-L1 Using a Rapid Point of Care Test for Predicting Head Computed Tomography Lesions After Mild Traumatic Brain Injury in a Dutch Multi-Center Cohort. J Neurotrauma. 2024;41(13-14):e1630-e40.
49. Foks KA, van den Brand CL, Lingsma HF, van der Naalt J, Jacobs B, de Jong E, et al. External validation of computed tomography decision rules for minor head injury: prospective, multicentre cohort study in the Netherlands. BMJ. 2018;362:k3527.
50. Curran JM, Onions K, Watts J, Rana A, Hughes E, Allison J, et al. Ubiquitin C-terminal hydrolase-L1 and glial fibrillary acidic protein tandem brain biomarker test in the prediction

of CT evident brain injury: A prospective evaluation in the emergency department. Ann Clin Biochem. 2025:45632251326483.
51. Harris M, Yue JK, Jain S, Sun X, Puccio AM, Gardner RC, et al. Effect of blood alcohol on the diagnostic accuracy of glial fibrillary acidic protein and ubiquitin carboxy-terminal hydrolase L1 for traumatic intracranial hemorrhage: A TRACK-TBI study. Acad Emerg Med. 2025.
52. Haacke EM, Duhaime AC, Gean AD, Riedy G, Wintermark M, Mukherjee P, et al. Common data elements in radiologic imaging of traumatic brain injury. J Magn Reson Imaging. 2010;32(3):516-43.
53. Iverson GL, Minkkinen M, Karr JE, Berghem K, Zetterberg H, Blennow K, et al. Examining four blood biomarkers for the detection of acute intracranial abnormalities following mild traumatic brain injury in older adults. Front Neurol. 2022;13:960741.
54. Kopcinovic Milevoy L, Gabaj N, Lapić I, Rogić D, Oprea OR, Dobreanu M, et al. Exclusion of intracranial lesions in mild traumatic brain injury using glial fibrillary acidic protein and ubiquitin C-terminal hydrolase-L1: a European multicenter study. Eur J Emerg Med. 2025.
55. Ladang A, Vavoulis G, Trifonidi I, Calluy E, Karagianni K, Mitropoulos A, et al. Increased specificity of the "GFAP/UCH-L1" mTBI rule-out test by age dependent cut-offs. Clin Chem Lab Med. 2025;63(5):995-1003.
56. Lagares A, de la Cruz J, Terrisse H, Mejan O, Pavlov V, Vermorel C, et al. An automated blood test for glial fibrillary acidic protein (GFAP) and ubiquitin carboxy-terminal hydrolase L1 (UCH-L1) to predict the absence of intracranial lesions on head CT in adult patients with mild traumatic brain injury: BRAINI, a multicentre observational study in Europe. EBioMedicine. 2024;110:105477.
57. Lagares A, Castaño-Leon AM, Richard M, Tsitsopoulos PP, Morales J, Mihai P, et al. Variability in the indication of brain CT scan after mild traumatic brain injury. A transnational survey. Eur J Trauma Emerg Surg. 2023;49(3):1189-98.
58. Lapić I, Rogić D, Lončar Vrančić A, Gornik I. Exploratory analysis of glial fibrillary acidic protein and ubiquitin C-terminal hydrolase L1 in management of patients with mild neurological symptoms undergoing head computed tomography scan at the emergency department: a pilot study from a Croatian tertiary hospital. Lab Med. 2024;55(4):492-7.
59. Legramante JM, Minieri M, Belli M, Giovannelli A, Agnoli A, Bajo D, et al. Evaluation of GFAP/UCH-L1 biomarkers for computed tomography exclusion in mild traumatic brain injury (mTBI). Int J Emerg Med. 2024;17(1):164.
60. Li Y, Ding VY, Chen H, Zhu G, Jiang B, Boothroyd D, et al. Comparing blood biomarkers to clinical decision rules to select patients suspected of traumatic brain injury for head computed tomography. The Neuroradiology Journal. 2023;36(1):68-75.
61. Oris C, Bouillon-Minois JB, Kahouadji S, Pereira B, Dhaiby G, Defrance VB, et al. S100B vs. "GFAP and UCH-L1" assays in the management of mTBI patients. Clin Chem Lab Med. 2024;62(5):891-9.
62. Oris C, Khatib-Chahidi C, Pereira B, Bailly Defrance V, Bouvier D, Sapin V. Comparison of GFAP and UCH-L1 Measurements Using Two Automated Immunoassays (i-STAT(®) and Alinity(®)) for the Management of Patients with Mild Traumatic Brain Injury: Preliminary Results from a French Single-Center Approach. Int J Mol Sci. 2024;25(8).
63. Papa L, Ladde JG, O'Brien JF, Thundiyil JG, Tesar J, Leech S, et al. Evaluation of Glial and Neuronal Blood Biomarkers Compared With Clinical Decision Rules in Assessing the Need for Computed Tomography in Patients With Mild Traumatic Brain Injury. JAMA Netw Open. 2022;5(3):e221302.
64. Puravet A, Oris C, Pereira B, Kahouadji S, Gonzalo P, Masson D, et al. Serum GFAP and UCH-L1 for the identification of clinically important traumatic brain injury in children in France: a diagnostic accuracy substudy. The Lancet Child & Adolescent Health. 2025;9(1):47-56.

65. Trivedi D, Forssten MP, Cao Y, Ismail AM, Czeiter E, Amrein K, et al. Screening Performance of S100 Calcium-Binding Protein B, Glial Fibrillary Acidic Protein, and Ubiquitin C-Terminal Hydrolase L1 for Intracranial Injury Within Six Hours of Injury and Beyond. Journal of Neurotrauma. 2024;41(3-4):349-58.
66. Welch RD, Bazarian JJ, Chen JY, Chandran R, Datwyler SA, McQuiston B, et al. A high-performance core laboratory GFAP/UCH-L1 test for the prediction of intracranial injury after mild traumatic brain injury. Am J Emerg Med. 2025;89:129-34.
67. Lagares A, Payen JF, Biberthaler P, Poca MA, Mejan O, Pavlov V, et al. Study protocol for investigating the clinical performance of an automated blood test for glial fibrillary acidic protein and ubiquitin carboxy-terminal hydrolase L1 blood concentrations in elderly patients with mild traumatic BRAIN Injury and reference values (BRAINI-2 Elderly European study): a prospective multicentre observational study. BMJ Open. 2023;13(7):e071467.
68. Lorton F, Lagares A, de la Cruz J, Mejan O, Pavlov V, Sapin V, et al. Performance of glial fibrillary acidic protein (GFAP) and ubiquitin carboxy-terminal hydrolase L1 (UCH-L1) biomarkers in predicting CT scan results and neurological outcomes in children with traumatic brain injury (BRAINI-2 paediatric study): protocol of a European prospective multicentre study. BMJ Open. 2024;14(5):e083531.
